# Influence of *Tenebrio molitor* Meal Inclusion (25–45%) on Clinical and Behavioral Responses in Laboratory Rat Feeding Trial

**DOI:** 10.3390/ani16111623

**Published:** 2026-05-26

**Authors:** Remigiusz Gałęcki, Beata Wesołowska, Nils Th. Grabowski

**Affiliations:** 1 Department of Veterinary Prevention and Feed Hygiene, Faculty of Veterinary Medicine, University of Warmia and Mazury in Olsztyn, 10-719 Olsztyn, Poland; 2 Department of Pathophysiology, Forensic Veterinary Medicine and Administration, Faculty of Veterinary Medicine, University of Warmia and Mazury in Olsztyn, 10-719 Olsztyn, Poland; 3 Institute for Food Quality and Food Safety, University of Veterinary Medicine Hannover (TiHo), 30173 Hannover, Germany

**Keywords:** edible insects, yellow mealworm, model organism, nutritional tests, novel food, animal feed, physiological examination, food and feed safety, behavioral research

## Abstract

Although *Tenebrio molitor* is increasingly explored as a feed ingredient, uncertainty remains regarding the effects of high dietary inclusion levels in model animals. Therefore, this study evaluated physiological and behavioral responses to graded inclusion levels of *T. molitor* meal (25–45%) in the diets of adult female Wistar rats. The assessed outcomes comprised (i) clinical parameters, (ii) food and water intake, (iii) hematology and serum biochemistry, and (iv) behavior. Across the insect-fed groups, no unintended weight loss or excessive gain was observed; instead, animals modulated their feed intake in a manner consistent with maintaining a relatively stable growth trajectory, without signs of hyperphagia suggestive of nutrient deficiency or anorexia indicative of adverse effects under study conditions. Diet acceptance remained high throughout the experiment, with no feed refusal or behavioral evidence of aversion that might be expected in response to poor palatability or discomfort. Heart rates and respiratory rates remained age-appropriate, showing typical temporal variability. Significant between-group differences were observed in RDW, Retic (%), NEU (%, K/μL), EOS, PLT, and PCT (*p* < 0.05), while serum analyses revealed significant differences in AST, total bilirubin, creatinine, phosphorus, and BUN (*p* < 0.05). A moderate negative correlation between individual feed intake and body weight was additionally identified in selected groups. The results of behavioral testing reveal that the dietary inclusion of *T. molitor* meal at levels up to 45% did not reduce spontaneous activity or elicit anxiety-like responses, and exploratory behavior appeared to be preserved. Overall, the present findings indicate that the gradual incorporation of *T. molitor* meal at high inclusion levels supported normal growth, physiological function, and behavior in rats under the conditions studied.

## Simple Summary

The increasing use of edible insects, such as yellow mealworm, in animal nutrition raises questions about the safety of high dietary inclusion levels. This study examined the effects of incorporating 25–45% insect meal into the diets of laboratory rats to assess potential effects on health and behavior. The assessment included clinical status, feed and water intake, blood and biochemical parameters, and behavioral responses. Rats fed the insect-based diet maintained stable growth without abnormal weight gain or loss. The animals adjusted their food intake accordingly, suggesting an equal nutrient supply with no overt adverse effects detected during the study. No signs of feed refusal or aversion were observed, and heart and respiratory rates were within age-related reference ranges. Some hematological and biochemical parameters differed significantly between groups. Behavioral tests did not reveal decreased activity, anxiety responses, or impairments in exploration. Overall, high levels of yellow mealworm meal supported normal physiological and behavioral development under the conditions tested. These findings support the potential use of yellow mealworm meal as a promising feed ingredient under the conditions tested.

## 1. Introduction

Insects have gained increasing attention as sustainable protein sources in food and feed due to their favorable feed conversion efficiency, relatively low land and water requirements, and potential for upcycling low-value substrates [1,2,3]. Among them, the yellow mealworm (*Tenebrio molitor*) is particularly notable because larvae provide substantial amounts of protein and lipids as well as bioactive components [4,5,6]. Recent compositional assessments of commercially reared *T. molitor* confirm a high content of protein and unsaturated fatty acids, supporting its suitability for monogastric nutrition [5]. The chemical composition and nutritional value of *T. molitor* larvae may vary depending on the culture conditions, and especially on the composition of the substrate used during larval development [7,8]. Ontogenetic profiling has also shown significant variability in protein, fat, and chitin content across developmental stages [4], which may influence physiological responses in consumers. Safety assessments carried out by the European Food Safety Authority (EFSA) consider dried, frozen, and UV-treated preparations of whole *T. molitor* larvae to be safe for the proposed uses and inclusion levels, while noting a potential risk of allergic reactions in susceptible individuals, particularly those allergic to crustaceans and house dust mites [9,10].

In addition to their sustainability and compositional attractiveness, accumulating evidence suggests that insect meals can modulate host physiology. In rodent models, *T. molitor* meals or extracts have demonstrated anti-lipidemic and hypolipidemic effects and improvements in metabolic profiles, potentially mediated through alterations in hepatic lipid synthesis and phospholipid remodeling [11,12]. In adult female Wistar rats, diets containing 35% *T. molitor* meal altered gut microbiota structure without apparent dysbiosis, consistent with functional adaptation to high-protein, chitin-rich substrates [13]. However, despite a growing body of research in poultry, fish, and swine, relatively few studies in rats have characterized systemic hematology and serum biochemistry, as well as growth performance, feed intake, behavior, and clinical indices at varying inclusion levels of *T. molitor* meal. Rats are an important model organism and play a key role in the development and evaluation of pharmaceuticals, innovations, and gastrointestinal research relevant to human and veterinary medicine, including monogastric animals [14,15,16,17,18]. In this context, such studies provide a controlled and ethically appropriate preliminary system for assessing dietary tolerance, physiological responses, and potential upper inclusion limits before use in the target species. This approach is consistent for the initial screening of new feed ingredients.

Edible insects typically exert neutral or beneficial effects on animal health and performance when diets are formulated to be isoenergetic and isonitrogenous. In poultry, edible insect meals can maintain or improve growth performance and feed efficiency, while carcass characteristics remain largely unaffected. Some studies have also reported favorable changes in blood lipid profiles and liver enzyme activity at low inclusion levels of insect meals, consistent with improved metabolic status in poultry [19,20,21,22]. In pigs, black soldier fly (*Hermetia illucens*) meals consistently support growth comparable to that observed with conventional protein sources. They may also improve gut integrity, for example by increasing the intestinal villus-to-crypt ratio, as well as by stimulating innate defense mechanisms attributed to short-chain fatty acids, antimicrobial peptides, and chitin-derived immunomodulatory effects [23,24,25]. Controlled trials and analyses in companion animals have shown that insect-based diets are digestible and generally well tolerated, with hematological and biochemical parameters remaining within physiological ranges and fecal quality unaffected at typical inclusion levels [26,27,28,29]. These data suggest that insect meals may affect performance and health indicators in all taxa, while the magnitude and direction of the response depend on species, composition, processing method (such as defatting), and amino acid supplementation.

High-protein dry diets, whether based on rendered animal meals or novel protein sources, may pose challenges to extrusion and pellet quality, which in turn can affect palatability and intake [30,31,32]. Recent pet food engineering studies have shown that the inclusion of insect meal affects the rheological properties, expansion, density, hardness, and stiffness of extruded products. In formulations with increasing insect meal content, pellets became softer and more deformable, while nutrient profiles remained within the target ranges, although moderate dilution of essential amino acids was observed and could be adjusted through supplementation [31,32]. These processing-related textural changes warrant investigation into whether higher proportions of insect meal also affect intake patterns, hydration status, and behavior in vivo.

*Tenebrio molitor* larvae meal has been extensively investigated as an alternative protein source for monogastric animals [29]; however, most in vivo experiments involve low or moderate levels of dietary intake. In poultry, the most commonly estimated range was 2–15%, and in pigs, 5–10%. Importantly, in the available studies, exposure levels were often defined as a percentage of protein source replacement, rather than as the absolute share of *T. molitor* in the complete feed mixture. For this reason, data for very high inclusion levels, especially those of 25–45% of the total diet, remain limited and difficult to compare with the prevailing literature. Even more recent studies in farmed species typically do not exceed a 20% *T. molitor* contribution, and in the rat model, a 35% contribution has been primarily described in studies focused on the microbiota. In this context, the 45% level should not be considered a direct practical recommendation, but rather a deliberately high exposure level to assess the margin of tolerance. Also, key uncertainties remain regarding the effects of its high dietary inclusion levels in model animals. Most laboratory studies in rats focus on feed safety or gut microbiota at single inclusion levels or on feeds containing extracts, rather than evaluating graded dietary incorporation, while simultaneously monitoring hematology, serum biochemistry, intake, behavior, and clinical symptoms [12,13]. The interpretation of research findings is further complicated by formulation variables such as defatting, lipid content, and chitin levels, all of which can alter digestibility and immune signaling. Chitin, in particular, has been associated with changes in lipid metabolism and innate immune responses in rodents [33,34]. However, the systemic implications of such variations in rats are unclear. Available studies show that *T. molitor* can affect growth, microbiota, metabolic parameters, and selected histological features, but endpoints are usually examined fragmentarily. Poultry studies have sometimes combined hematology with intestinal morphology and histopathology, and studies on obese mice have combined metabolism, histology, and microbiota. However, no study has been identified that combines clinical/physiological, behavioral, hematological, and biochemical assessments in a single experiment on laboratory animals with a very high, graded share of *T. molitor* in the diet. Such integration is particularly valuable in tolerance studies, as it allows for the detection of both functional changes and potential organ symptoms. Safety reviews have also highlighted the potential for allergenicity, emphasizing the importance of integrated physiological assessments [9,35,36]. To address these gaps, the aim of the present study was to evaluate the physiological and behavioral effects of gradual incorporation of *T. molitor* meal (25–45%) into the diet of adult female Wistar rats. The assessed outcomes included (i) clinical parameters, (ii) feed and water intake, (iii) hematological profile and serum biochemistry, and (iv) behavior. The study was designed as a preliminary assessment of the safety margin and tolerability of graded, high-level inclusion of *T. molitor* meal. It was hypothesized that gradually increasing the level of inclusion, and in particular the highest inclusion level of 45%, would reveal potential clinical, biochemical, hematological, food-related, or behavioral responses under controlled feeding conditions.

## 2. Materials and Methods

### 2.1. Feed Preparation

Live *T. molitor* larvae were obtained from a domestic producer (Tenebria, Lubawa, Poland). To reduce this source of *T. molitor* variability, larvae used in the whole project were obtained from a single supplier throughout the study and were reared on a standard grain-based substrate consisting mainly of wheat bran supplemented with vegetables as a moisture source. Prior to processing, larvae were fasted for 72 h to empty the gut and were subsequently mechanically sifted to remove residual feed, frass, shed exoskeletons, and any dead or pupated individuals. The larvae used for meal production were last-instar larvae (approximately 10–12 weeks old). They were euthanized by thermal blanching at 90 °C, dried, and finely milled to obtain a full-fat, non-dechitinized meal. The experimental diets were prepared in dry form as oval pellets with a diameter of approximately 15 mm. Six dietary treatments were prepared: insect-based diets containing 25%, 30%, 35%, 40% and 45% full-fat, non-dechitinized *T. molitor* meal, and a poultry-based diet containing 35% commercially available poultry meal. Experimental diets were produced in 50 kg batches: the 25%, 30%, 35%, 40%, and 45% *T. molitor* diets contained 12.5, 15.0, 17.5, 20.0, and 22.5 kg of insect meal per batch, respectively, and the 35% poultry-meal reference would contain 17.5 kg poultry meal. A poultry meal-based diet was used as the reference diet, representing a conventional animal protein formulation commonly used in animal nutrition, particularly in diets for companion animals with diet-related enteropathies. This reference diet was chosen to provide a practical and industry-relevant comparison. The inclusion range was selected to provide a graded series of high *T. molitor* meal concentrations, enabling evaluation of dose-related physiological and behavioral effects under conditions in which *T. molitor* served as a major protein source, while remaining technologically feasible for dry- dog food manufacture. The 25–45% inclusion range was selected to represent technologically realistic use levels of animal meals in pet food manufacturing and to test the tolerance of rats to progressively high dietary concentrations of *T. molitor* meal. Inclusion range is also consistent with previous insect meal feeding studies [37]. All remaining components were identical across the treatments. The details of some technological processes constitute the subcontractor’s intellectual property. According to the provided information, the production process involved dry-feed extrusion using industrial pet-food processing methods. After extrusion, the feed granules were dried at approximately 105 °C for about 16 min and approximately 6 min of cooling to obtain a shelf-stable dry product. The complete diet formulations are protected under patent No. 247885. The diets were originally developed as hypoallergenic options for dogs with food-responsive enteropathies [32]. The experimental diets (25%, 30%, 35%, 40%, 45% *T. molitor* meal, and 35% poultry meal) were formulated to be approximately isoenergetic and isonitrogenous at the proximate level. On a dry matter basis, crude protein ranged from 25.73% to 25.90%, crude fat from 13.56% to 14.07%, crude ash from 5.36% to 5.73%, crude fiber from 5.16% to 5.29%, and carbohydrates from 42.01% to 43.15% across the 25–45% inclusion range. The chitin content was estimated based on data for *T. molitor* included in the EFSA Panel on Nutrition publication [9]. For dried and powdered *T. molitor* (#23–30 [9]), the average amount of chitin was calculated to be 6.5% on a dry matter basis. Under the adopted assumption, the estimated chitin content in insect diets was 1.63, 1.95, 2.27, 2.60, and 2.93%, respectively, for diets containing 25%, 30%, 35%, 40%, and 45% full-fat *T. molitor* meal. These values should be interpreted as literature estimates and not direct analytical determinations of the prepared diets. Gross energy (GE) and metabolizable energy (ME) values were very similar in all five diets, indicating that changes in macronutrient composition maintained almost unchanged caloric density across all treatments. GE showed only minimal variation, from 452.14 kcal/100 g in the diet containing 25% *T. molitor* meal to 453.58 kcal/100 g in the diet with 40% inclusion, with a mean of 452.9 ± 0.6 kcal/100 g. Similarly, ME values varied only slightly, from 356.48 to 357.93 kcal/100 g, with a mean of 357.0 ± 0.6 kcal/100 g. In all formulations, the maximum difference observed was less than 1.5 kcal/100 g for both GE and ME [32]. Mean content (%) of crude protein, fat, ash, fiber, and carbohydrates on a dry matter basis in formulas used for adult female Wistar rats feeding can be found in Supplementary Materials, Table S1. Detailed information on nutritional composition is provided in feed design study [32].

### 2.2. Laboratory Animals and Experimental Procedure

The study was designed and reported in accordance with the ARRIVE guidelines. Ethical approval was granted by the Local Ethics Committee (decision No. 31/2022 of 18 May 2020). All procedures complied with Polish legislation (Act of 15 January 2015 on the protection of animals used for scientific or educational purposes, Journal of Laws of 2015, item 266) and with Directive 2010/63/EU of the European Parliament and of the Council on the protection of animals used for scientific purposes. All procedures were conducted in accordance with the 3R principles (Replacement, Reduction, Refinement) [38,39]. The experimental model comprised adult female Wistar rats, 12 months of age, sourced from a single breeding colony. During a preliminary phase, the rats were habituated to handling and the personnel involved in the experiment. Animals underwent a 14-day acclimation period before dietary interventions.

Rats were housed in a dedicated laboratory animal facility under controlled conditions. Each cage had a floor area of ≥800 cm^2^ and internal height of ≥18 cm. Room temperature was maintained at 22 ± 2 °C, relative humidity at 60 ± 5%, and a 12:12 h light–dark cycle was applied with approximately 65 lx during the light phase. Animals were housed individually in transparent cages that enabled visual contact with other rats in order to support behavioral needs. Following acclimation, rats were randomly divided into seven groups and maintained on their respective diets for 60 days: Group A (control group)—standard laboratory rat diet (Labofeed H Standard, Wytwórnia Pasz “Morawski”, Żurawia, Warszawa, Poland); Group B (reference group)—poultry meal-based diet; Group C—diet containing 25% *T. molitor* meal; Group D—diet containing 30% *T. molitor* meal; Group E—diet containing 35% *T. molitor* meal; Group F—diet containing 40% *T. molitor* meal; Group G—diet containing 45% *T. molitor* meal.

Each group consisted of five animals (*n* = 5). A power analysis was considered for a between-group analysis of variance (ANOVA) design. With seven groups, *n* = 5, α = 0.05, and a statistical power of 0.80, the study was able to detect large to very large effects, corresponding approximately to Cohen’s *f* = 0.69 or *η*^2^ = 0.32. Therefore, the study should be interpreted as exploratory and primarily suitable for detecting main effects of diet. Randomization was performed using a computerized random generator, which ensured unbiased assignment of animals to experimental groups. The study had a single-blind design as the animal handlers, personnel conducting clinical and behavioral assessments, and laboratory personnel performing hematological and biochemical analyses were blinded to the group allocation. Due to practical and organizational constraints of the study, the principal investigator and the person responsible for data storage were aware of the group allocation. Group A served as a reference point for physiological parameters under conventional feeding conditions. Group B was included as a practical comparison diet based on poultry meal, based on animal protein, and its purpose was to distinguish effects specifically attributed to *T. molitor* meal from effects generally associated with the use of a concentrated animal protein component in the diet formulation.

Feed and water were provided ad libitum. The experimental diets were offered ad libitum in quantities exceeding the expected voluntary intake to model a nutritional overload scenario and to assess tolerance to high dietary inclusion levels of *T. molitor* meal. Feed intake was measured daily. To facilitate data presentation, the average daily feed intake per animal was calculated at weekly intervals, and the last measurement period covered the final four days of week 9. Water intake was measured and replenished every three days. The animals were weighed at weekly intervals, and the body condition score (BCS) was assessed during weighing based on the scale developed by Gades and Murray [40]. Fecal consistency was evaluated daily using the scale proposed by de Queiroz et al. [41]. Heart rate and respiratory rate were measured weekly using a digital stethoscope.

### 2.3. Hematology and Blood Biochemistry

At the end of the experimental period, rats were euthanized by isoflurane overdose, and blood was collected by cardiac aspiration. For hematological analysis, whole blood was processed according to the manufacturer’s recommendations and analyzed using an IDEXX ProCyte Dx (IDEXX Laboratories Inc., Westbrook, ME, USA) automated hematology analyzer. The following parameters were determined: red blood cell count (RBC, M/μL), hematocrit (HCT, %), hemoglobin (HGB, g/dL), mean corpuscular volume (MCV, fL), mean corpuscular hemoglobin (MCH, pg), mean corpuscular hemoglobin concentration (MCHC, g/dL), red cell distribution width (RDW, %), reticulocyte percentage (Retic, %), reticulocyte count (Retic, K/μL), white blood cell count (WBC, K/μL), neutrophils (NEU, % and K/μL), lymphocytes (LYM, % and K/μL), monocytes (MONO, % and K/μL), eosinophils (EOS, % and K/μL), basophils (BASO, % and K/μL), platelet count (PLT, K/μL), mean platelet volume (MPV, fL), platelet distribution width (PDW, fL), and plateletcrit (PCT, %).

For serum biochemistry, a portion of blood was allowed to clot, and serum was separated by centrifugation using standard laboratory procedures. Selected biochemical parameters were measured using an IDEXX Catalyst One analyzer (IDEXX Laboratories Inc., Westbrook, ME, USA): glucose (mmol/L), triglycerides (TG, mmol/L), alanine aminotransferase (ALT, U/L), aspartate aminotransferase (AST, U/L), total bilirubin (BIL T, μmol/L), alkaline phosphatase (ALP, U/L), creatinine (CREA, μmol/L), urea (UREA, mmol/L), albumin (ALB, g/L), creatine kinase (CK, U/L), calcium (CALC, mmol/L), phosphorus (PHOS, mmol/L), blood urea nitrogen (BUN, mg/dL), and cholesterol (CHOL, mmol/L). These serum biochemical indicators were selected to provide an integrated assessment of the overall nutritional and metabolic status, as well as organ function in the experimental rats.

### 2.4. Behavioral Test Procedures

All behavioral tests were conducted during the rats’ active period (dark phase) following a nine-week feeding trial. Multiple complementary behavioral studies were used to improve sensitivity. Behavioral testing was performed sequentially in a single session before final samples were collected to minimize repetitions across days and to standardize testing conditions at the established experimental endpoint. To standardize motivational state for the nutritional choice test, rats were fasted for approximately 12 h prior to behavioral assessment. This approach was chosen to ensure a uniform physiological state across animals, to avoid repeated handling and repeated anticipatory stress on multiple days, and to allow completion of all behavioral assessments before post-mortem sampling.

#### 2.4.1. Open-Field Test

The open-field test was used to assess locomotor activity and exploratory or anxiety-related behavior in a novel environment. At the beginning of the trial, each rat was gently placed in a corner of a square open-field arena with a softly lit central area and was allowed to explore freely for three minutes. During this time, activity was recorded and specific behavioral parameters were measured. The total distance traveled by each rat in the arena was tracked as an indicator of locomotor activity. In addition, behaviors associated with exploration or anxiety were observed and quantified, including: (i) central area entries, defined as the number of times the rat entered the designated central zone of the arena, reflecting willingness to explore open space; (ii) rearing, defined as the frequency of vertical exploration when the rat stood on its hind legs; (iii) grooming, defined as the number of self-grooming episodes observed; and (iv) freezing, defined as the number of episodes of complete immobility. All behaviors were recorded by an observer and a tracking system according to standard open-field protocols. After a three-minute session, the rat was removed from the arena and prepared for the following test. The arena was cleaned between tests to eliminate any residual scent cues.

#### 2.4.2. Eight-Arm Radial Maze

An eight-arm radial maze (Olton-Samuelson type apparatus) was used to assess rats’ exploration of multiple arms radiating from a central platform. Each rat was placed in the central hub of the radial maze apparatus at the beginning of the trial. The maze consisted of eight arms extending outward from the center at equal angles. In this version of the task, there were no feed rewards; the rat was simply allowed to freely explore the maze. Each trial lasted five minutes, during which arm entries were recorded. An arm entry was defined as the rat moving with all four paws crossing the threshold of an arm. The sequence and number of arm visits were recorded for each animal. The main outcome measures were: (i) total arm entries, including repeated entries into the same arm; (ii) unique arm entries, defined as the number of different arms (out of 8 arms) entered at least once during the trial, reflecting the breadth of exploration; (iii) rearing; (iv) grooming; and (v) freezing. These parameters were used to assess overall activity in the maze and the animal’s tendency to explore new arms versus revisiting previously entered arms. The radial maze apparatus was cleaned between trials to remove odor cues. The eight-arm maze is a well-established tool for studying spontaneous spatial exploration, locomotor activity, and arm-visit patterns.

#### 2.4.3. Nutritional Choice Test

A feed choice test was conducted to assess food preference and motivation. Three different diets were presented simultaneously in an open arena. Identical glass containers (small open-topped jars) were placed in three separate corners of the arena, each containing one of the following diets: R—insect-based diet (an experimental high-protein insect-based diet assigned to one of the study groups; groups A and B received feed supplemented with 35% insect meal); P—poultry meal-based diet; and S—standard laboratory rat diet. Each rat (regardless of group) was placed in the arena and allowed to move freely and explore the available feed options for three minutes. The positions of the R, P, and S feed containers were randomized for each animal to eliminate location-related bias. During the test, the number of approaches to each feed source was recorded. An approach was defined as a rat moving towards a feed container and exhibiting exploratory behavior, such as sniffing or touching the feed. The following measures were derived from the observations: the total number of approaches to any feed item as an indicator of overall exploratory activity and motivation during the test; and the number of approaches to each feed type, which enabled comparison of relative interest. All rats were tested under the same conditions. Instances of actual feeding behavior were recorded qualitatively; however, given the short duration of the trial, the test primarily assessed approach and choice behavior rather than consumption. After each trial, the feed containers were removed, and spilled feed was cleared before the following test. This test design provided insight into the rats’ feed preferences, including responses to novel versus familiar diets, and overall activity in a competitive feeding scenario.

### 2.5. Statistical Analysis

The assumptions of normality and homogeneity of variances were examined before parametric testing. The normality of residuals was evaluated with the Shapiro–Wilk test, and homogeneity of variances was assessed with Levene’s test. As both assumptions were met (*p* > 0.05), the data were analyzed using one-way analysis of variance (ANOVA) or repeated-measures ANOVA, where the group was the independent factor. The measured physiological and behavioral parameters served as dependent variables. When a significant effect was detected in ANOVA, Tukey’s HSD test was applied for pairwise comparisons. The analyses and descriptive statistics were performed using Statistica 13.3 (TIBCO Software Inc., Palo Alto, CA, USA). The relationship between heart rate and respiratory rate was examined in each experimental group using repeated-measures correlation (rmcorr package 0.7.0) in the R statistical environment (RStudio 4.3.1). This method was also used to analyze the relationship between body mass and mean daily feed intake. Statistical significance was set at *p* < 0.05.

## 3. Results

### 3.1. Clinical Parameters

All groups differed significantly in mean daily feed intake over the nine-week period (ANOVA group effect: df = 6; F = 368.5; *p* < 0.001). Group A consistently exhibited the lowest feed intake, averaging only about 8.5 ± 1.0 g/day throughout the study. Total feed intake was significantly higher in groups B–G, where mean intake ranged from around 15 to 18 g/day. This difference was evident from week 1, when feed intake in group A (7.9 g) was significantly below that noted in the other groups (approx. 18–19 g). Throughout the experiment, feed intake in group A remained significantly lower than in the remaining groups at every time-point (Tukey HSD post hoc, *p* < 0.05).

**Figure 1:**
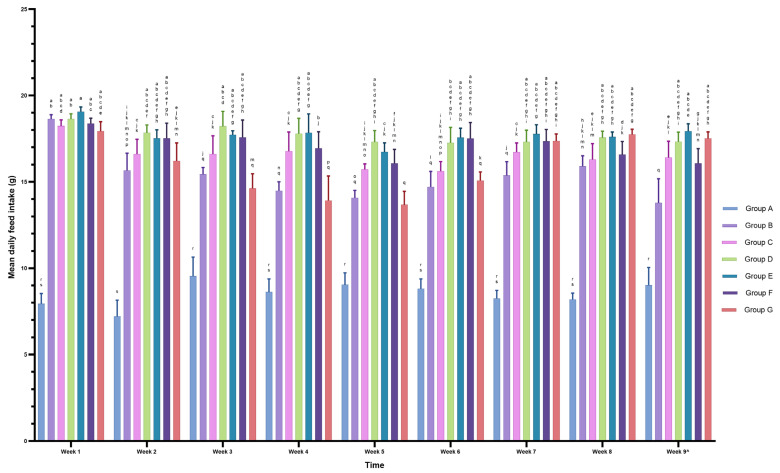
Weekly mean daily feed intake in adult female Wistar rats over the 9-week feeding trial (mean ± SD) across dietary groups. Legend: Different letters (a–s) denote statistically homogeneous groups. Statistical significance was determined using Tukey’s HSD post hoc test after analysis of variance; all possible pairwise comparisons between groups were tested. ^—Mean daily feed intake was calculated based on the last 4 days of the experiment. A—standard laboratory rat diet, B—poultry meal-based diet, C—25% *T. molitor* meal, D—30% *T. molitor* meal, E—35% *T. molitor* meal, F—40% *T. molitor* meal, and G—45% *T. molitor* meal.

A highly significant effect of time on feed intake (df = 8; F = 30.27, *p* < 0.001) as well as a significant group vs. week interaction (df = 48; F = 8.27, *p* < 0.001) were also observed, which indicates that temporal variations in feed intake differed between groups. Most groups showed an initial decrease in daily intake after the first week, but to varying degrees. Groups B and G exhibited the most pronounced decreases. In group B, mean intake declined from 18.6 ± 0.3 g/day in week 1 to 14.1 ± 0.4 g/day in week 5. A similar pattern was observed in group G, where feed intake decreased from 17.9 ± 0.6 g in week 1 to only 13.7 ± 0.8 g in week 5, representing a 25% reduction, before increasing to 17.5 ± 0.4 g in week 9. In contrast, groups D and E maintained relatively stable and high feed intake throughout the study, with only minor fluctuations. In group D, feed intake decreased from 18.6 g in week 1 to around 17–18 g in subsequent weeks, ending at 17.3 ± 0.5 g in week 9. Group E had a similarly high intake at 19.1 g in week 1 and 17–18 g in the following weeks. Groups C and F showed intermediate patterns, with small decreases of approximately 2–3 g after week 1 and partial recovery thereafter. By week 9, all high-intake groups (B–G) approached the 16–18 g range, while group A remained markedly lower at 9.0 ± 1.0 g. Weekly mean daily feed intake across dietary groups are presented in Figure 1.

Water intake differed significantly between groups (df = 6; F = 4.23, *p* = 0.0038). Water intake was highest in group B (30 ± 4 mL/day) and lowest in group E (22 ± 3 mL/day), and the remaining groups showed intermediate values, ranging from approximately 25 to 28 mL/day. A highly significant effect of time was also observed (df = 19; F = 92.41, *p* < 0.0001), together with a significant group vs. time interaction (df = 114; F = 3.63, *p* < 0.0001), indicating different temporal patterns of water intake between groups. All groups showed an early peak in water intake around day 9, followed by a mid-study decline that was most pronounced in group E, and a subsequent recovery phase in several groups. By day 60, water intake converged across groups, and comparable levels were observed in all groups at the end of the study.

**Figure 2:**
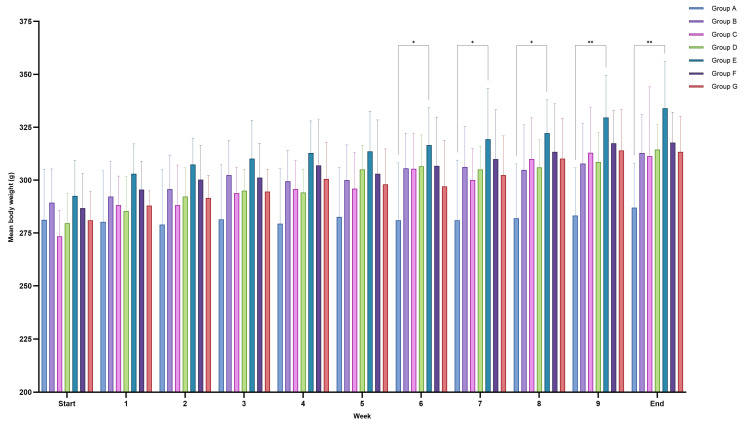
Weekly body weight changes in adult female Wistar rats during the 9-week feeding trial (mean ± SD) across dietary groups. Legend: A—standard laboratory rat diet, B—poultry meal-based diet, C—25% *T. molitor* meal, D—30% *T. molitor* meal, E—35% *T. molitor* meal, F—40% *T. molitor* meal, and G—45% *T. molitor* meal; * *p* < 0.05; ** *p* < 0.01. Statistical significance was determined using Tukey’s HSD post hoc test after analysis of variance; all possible pairwise comparisons between groups were tested.

At baseline (week 0), mean body weight was similar across groups, ranging from approximately 273 ± 11 g (group C) to 293 ± 15 g (group E). No significant differences in baseline body weight were determined between groups (ANOVA main group effect: df = 6; F = 1.63; *p* = 0.175). During the nine-week experiment, body weight increased in all groups (significant effect of time: df = 10; F = 59.15, *p* < 0.0001), but the magnitude of weight gain differed between groups (group vs. time interaction: df = 60; F = 2.03; *p* < 0.001). All treatment groups, except group A, showed significant weight gain, whereas body weight in group A remained relatively stable. From baseline to week 9, mean body weight in group A increased by only 6 g (from 281 ± 21 g to 287 ± 19 g), whereas the increase in the remaining groups approximated 25–40 g. At the end of the study, group E reached the highest mean body weight (334 ± 20 g), which was significantly greater than that in group A (287 ± 19 g, *p* < 0.01). Final body weights in groups B, C, D, F, and G were intermediate (approx. 313–318 g) and did not differ significantly from groups A and E in pairwise comparisons (Tukey’s post hoc test, *p* > 0.05). These results indicate that although body weight increased over time in all groups, group A exhibited only minimal weight gain, resulting in significantly lower final body weight relative to the other groups, particularly group E, which showed the highest weight gain. Weekly body weight changes are presented in Figure 2.

A statistically significant, moderate negative relationship between body weight and mean daily feed intake was determined in groups B (r_rm = −0.45; *p* = 0.003), C (r_rm = −0.41; *p* = 0.008), D (r_rm = −0.53; *p* < 0.001), and F (r_rm = −0.40; *p* = 0.010), indicating that intra-individual changes in feed intake over time were inversely related with changes in body weight. No significant correlations were found in groups A, E, or G (*p* > 0.05).

BCS values began to diverge from week 4 onwards, and group E rats gradually reached over-conditioning (BCS = 3.6). A similar trend was observed in group F, whereas group G showed a delayed but steady increase. Group D exhibited a moderate increase (BCS = 3.8), whereas groups B and C showed only minor changes. In group A, BCS remained stable at 3 at all time-points, indicating no change in body condition. No rats reached the maximum score of 5, and no cases of under-conditioning (BCS ≤ 2) were observed. Repeated-measures ANOVA revealed a significant group effect on BCS (df = 6; F = 2.91; *p* = 0.025), indicating differences in body condition between groups over time. The main effect of time (df = 8; F = 17.41; *p* < 0.001) was also significant, suggesting consistent temporal changes in BCS. However, the group vs. time interaction was not significant (df = 48; F = 1.36; *p* = 0.075), indicating that although overall changes occurred, their pattern was similar across groups.

Throughout the observation period, diarrhea scores remained at 0 in all rats in groups A–F on each day of the study, indicating a complete absence of diarrhea in these groups. In group G, diarrhea episodes were observed in individual animals on day 30 and between days 45–49, lasting 1–3 consecutive days. Non-zero scores were recorded seven times in total (2.3% of observations), whereas no diarrhea was recorded in groups A–F. All episodes resolved spontaneously on subsequent observation days.

**Figure 3:**
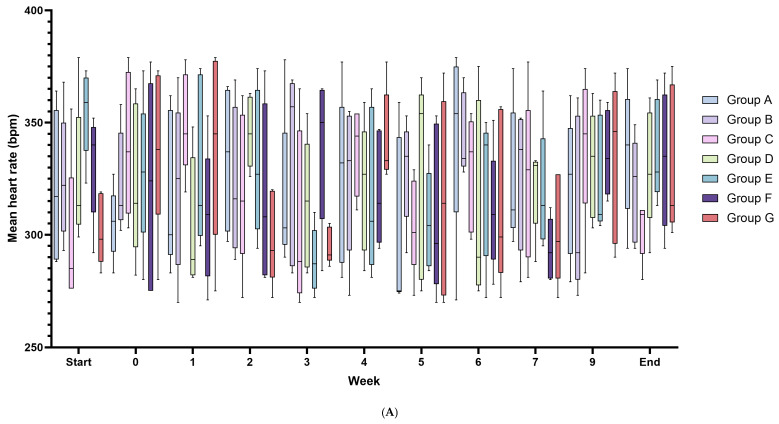

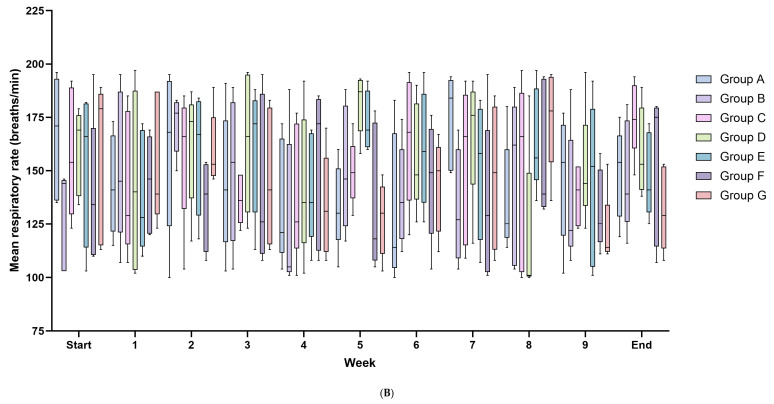
Weekly (**A**) mean heart rate and (**B**) mean respiratory rate in adult female Wistar rats during the 9-week feeding trial across dietary groups. Legend: The central line indicates the median, boxes represent the interquartile range (25th–75th percentiles), and whiskers denote the data range (up to 1.5 × IQR). A—standard laboratory rat diet, B—poultry meal-based diet, C—25% *T. molitor* meal, D—30% *T. molitor* meal, E—35% *T. molitor* meal, F—40% *T. molitor* meal, and G—45% *T. molitor* meal.

At all time-points, heart rate values were comparable across groups, with overlapping variability, but remained within physiological ranges. In group A, the baseline heart rate was approximately 258.5 ± 37.6 bpm. During the first four weeks, it increased to a mean value of 325.8 ± 39.5 at week 4, decreased to 294.2 ± 41.6 at week 5, and then increased rapidly to 354.5 ± 27.6 at the final measurement. In group B, the baseline heart rate was approximately 288.0 ± 27.8 and gradually increased, reaching a peak of 371.5 ± 24.3 at week 7, and then decreased to 284.7 ± 41.8 at the end of the study. Group C began at 283.3 ± 21.6, reached an early peak of 340.0 ± 41.9 at week 1, then declined and stabilized, with around 301.3 ± 27.0 at the final measurement. In group D, heart rate started at 309.7 ± 33.9, increased slightly, peaked at 362.0 ± 13.3 around week 7, remained high at 352.0 ± 28.2 at week 9, and concluded at 334.7 ± 36.2. Group E showed a baseline of 290.3 ± 11.7, rising mid-study (342.8 ± 17.4 at week 6 and 360.7 ± 20.8 at week 7), before declining to 335.7 ± 38.4 by the end of the study. Group F began at approximately 297.5 ± 28.2, reached a peak of 343.5 ± 43.0 at week 6, and ended at 338.3 ± 30.7. Group G had a baseline value of 305.8 ± 37.6, showed moderate fluctuations with a peak at approximately 338.7 ± 23.1 at week 8, and concluded at 308.5 ± 29.8. Overall, heart rates tended to increase mid-study in all groups. However, at the final measurement, heart rate values were elevated in groups A, D, E, and F (330–355), but returned to baseline levels in groups B and G. The statistical analysis confirmed that heart rate values did not change significantly over time or between groups. Repeated-measures ANOVA revealed no significant effect of time (df = 10; F = 1.2; *p* = 0.29), no significant main effect of group (df = 6; F = 0.23, *p* = 0.644), and no significant group vs. time interaction (df = 60; F = 1.11; *p* = 0.28). Data are presented in Figure 3A.

Overall, mean respiratory rates varied between groups, with overlapping confidence intervals, and the values remained within physiological ranges. At baseline, the mean respiratory rate was highest in group A (165.8 ± 28.7 breaths/min) and lowest in group B (128.2 ± 23.0 breaths/min). Across all 35 rats, the overall mean at baseline was 151.2 ± 30.4 breaths/min. By the final measurement, the mean respiratory rate was highest in group C (175.0 ± 17.4) and lowest in group G (132.0 ± 19.7). The values in groups A, B, and E ranged from around 147 to 149, and groups D and F showed intermediate values (158.8 and 152.6, respectively). Despite these differences, ANOVA revealed no significant group effect (df = 6; F = 2.43; *p* = 0.051), and no significant main effect of time (df = 10; F = 1.27, *p* = 0.248). The group vs. time interaction was not significant, either (df = 60; F = 0.95; *p* = 0.592). Weekly mean heart rate and mean respiratory rate are presented in Figure 3B.

No statistically significant correlations between respiratory rate and heart rate were observed in any group (all *p* > 0.05), and r_rm coefficients ranged from −0.20 to 0.07. The strongest trends were observed in group C (r_rm = −0.203; *p* = 0.15) and group E (r_rm = −0.16; *p* = 0.28). These results indicate that in the tested model, changes in heart rate and respiratory rate over time were largely independent of each other.

### 3.2. Hematology and Serum Biochemistry

As a complement to clinical observations, hematological and biochemical serum parameters were assessed to determine potential systemic and organ-specific effects of the diets. Among the measured hematological parameters, significant differences in RDW, Retic (%), NEU (both in % and K/all), EOS, PLT, and PCT (*p* < 0.05) were observed between groups. However, the groups did not differ significantly in the values of RBC, HCT, HGB, MCV, MCH, MCHC, Retic (K/μL), WBC, LYN, MONO, BASO, MPV, or PPDW (*p* > 0.05). Group F showed a significantly lower RDW (18.02 ± 1.39%) compared with the remaining groups (approx. 20–22%, *p* < 0.05). Specifically, RDW in group F was significantly lower than in groups A (20.4 ± 0.95%), B (21.1 ± 0.70%), C (21.84 ± 0.56%), D (21.64 ± 1.02%), E (21.3 ± 0.60%), and G (20.96 ± 1.20%) (Tukey HSD, *p* < 0.05). The percentage of reticulocytes differed significantly between groups (*p* < 0.01). Group B had the highest Retic (%) (5.14 ± 1.35%), which was significantly higher than in group E (3.10 ± 0.84%) and group F (2.62 ± 0.83%) (Tukey HSD, *p* < 0.05). In the remaining groups, Retic (%) values were intermediate without significant differences. An overall group effect was observed for NEU (ANOVA *p* = 0.039), but Tukey’s post hoc test did not reveal any specific pairwise differences at *p* < 0.05. Neutrophil (%) values tended to be highest in group E (43.3 ± 8.0%) and lowest in groups A and G (approx. 24–25%). Absolute NEU counts were significantly lower in group F (0.31 ± 0.28 K/μL) compared with group B (0.94 ± 0.25 K/μL) and group E (0.89 ± 0.20 K/μL) (*p* < 0.01). The remaining groups had intermediate NEU counts without significant differences. Eosinophil counts were low in all groups, but mean EOS count was slightly higher in group A (0.16 ± 0.13 K/μL) than in group F (0.02 ± 0.01 K/μL), and this difference was significant (*p* < 0.05). Significant differences in EOS counts were not observed in other groups. Marked differences were found in PLT counts and PCT values. Group B had significantly lower PLT counts (215.8 ± 170.3 K/μL) and PCT (0.19 ± 0.07%) compared with most other groups. In particular, PLT and PCT were significantly lower in group B than in groups A (625.0 ± 122.3; PCT 0.52 ± 0.08%), C (607.0 ± 77.8; PCT 0.52 ± 0.07%), D (667.2 ± 137.5; PCT 0.57 ± 0.07%), E (682.0 ± 89.9; PCT 0.56 ± 0.07%), and G (545.0 ± 229.0; PCT 0.46 ± 0.10%) (all *p* < 0.01). Platelet values in group F (458.4 ± 119.6; PCT 0.43 ± 0.09%) were lower than in the remaining groups except group B, but these differences were not statistically significant. Hematological data are presented in Table 1.

For serum biochemistry measurements, significant differences between groups were observed for AST, BIL T, CREA, PHOS, and BUN (*p* < 0.05). No significant differences between groups were found for glucose, TG, ALT, ALP, UREA, ALB, CK, Ca, and CHOL (*p* > 0.05). AST levels differed significantly between groups (*p* < 0.01). AST values were highest in group G (287.8 ± 63.7 U/L) and significantly higher than in group A (110.0 ± 57.3 U/L) and group E (168.0 ± 57.8 U/L) (Tukey HSD, *p* < 0.05). Group F (232.4 ± 60.9 U/L) also exhibited higher AST levels than group A (*p* < 0.05). In the remaining groups, mean AST levels were intermediate without significant differences. Total bilirubin levels were very low in all groups (means ranging from 0.00 to 0.04 µmol/L). Although an overall group effect was detected (ANOVA *p* = 0.036), caused by a slight increase in group G (mean 0.04 µmol/L) compared with the other groups (0.00 µmol/L), Tukey’s post hoc test did not reveal significant pairwise differences between groups. Group A had the lowest CREA concentration (22.6 ± 4.2 µmol/L), which was significantly lower than in groups C (31.6 ± 3.8), F (32.4 ± 3.8), and G (32.6 ± 6.1 µmol/L) (*p* < 0.01). CREA concentrations in the remaining groups (B, D, E) did not differ significantly from each other or from the other groups. PHOS concentrations showed a significant overall group effect (*p* < 0.05), with groups A and B displaying slightly higher mean values (1.6 mmol/L) than the remaining groups (1.1 mmol/L). However, no significant pairwise differences were determined in post hoc tests. BUN concentration was significantly higher in group A (3.40 ± 0.55 mg/dL) compared with group B (2.20 ± 0.45 mg/dL) and group D (2.20 ± 0.45 mg/dL) (*p* < 0.05). No significant differences were observed between the other groups. Biochemical data are presented in Table 2.

### 3.3. Open-Field Test

**Figure 4:**
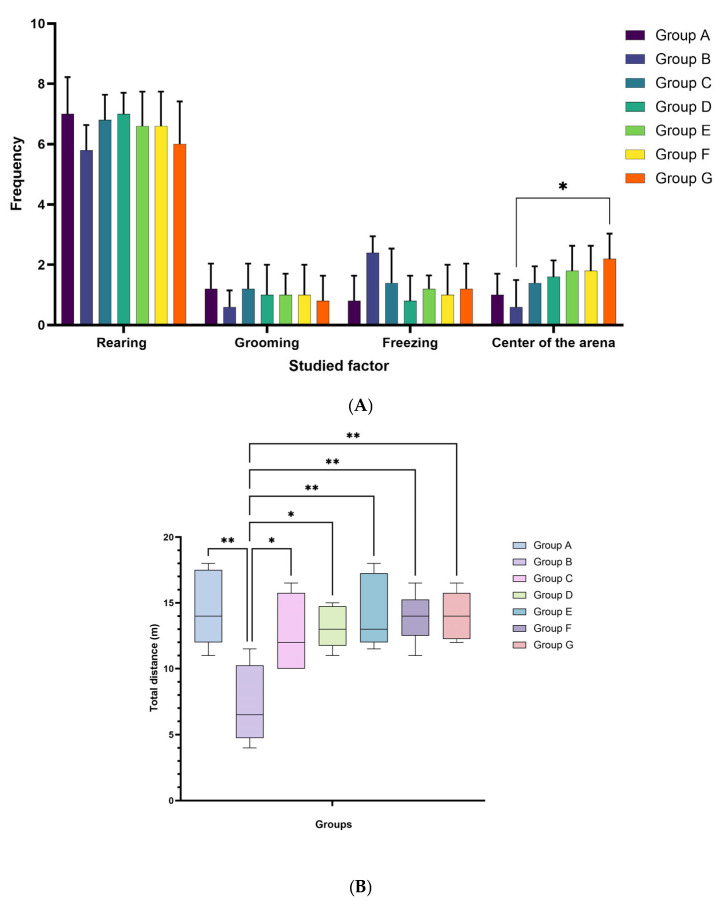
Open-Field Test outcomes in adult female Wistar rats across dietary groups A–G: (**A**) rearing, grooming, freezing, center activity, and (**B**) total distance traveled. Legend: (**A**) Columns represent mean ± SD values. (**B**) Box-and-whisker plots show the distribution of locomotor activity (m) recorded during the trial. The central line indicates the median, boxes represent the interquartile range (25th–75th percentiles), and whiskers denote the data range (up to 1.5 × IQR). * *p* < 0.05; ** *p* < 0.01. Statistical significance was determined using Tukey’s HSD post hoc test after analysis of variance; all possible pairwise comparisons between groups were tested. A—standard laboratory rat diet, B—poultry meal-based diet, C—25% *T. molitor* meal, D—30% *T. molitor* meal, E—35% *T. molitor* meal, F—40% *T. molitor* meal, and G—45% *T. molitor* meal.

In addition to physiological parameters, behavioral responses were assessed to determine whether dietary treatments influenced locomotor activity and exploratory behavior. There were no significant differences among groups in rearing frequency, grooming, or freezing behavior in the open field (ANOVA, *p* > 0.05 for all). All groups showed similar levels of rearing (6–7 rears per trial), minimal grooming (0–2 grooming episodes), and low numbers of freezing episodes (0–3 per trial). A significant group effect was observed for rat activity in the center of the arena (df = 6; F = 2.55; *p* = 0.043). Tukey HSD post hoc tests indicated that group B rats spent significantly less time in the center of the arena (0.6 ± 0.89) than group G animals (2.2 ± 0.84; *p* < 0.05). The remaining groups were characterized by intermediate values with no significant pairwise differences. Total distance traveled in the open field differed markedly between groups (df = 6; F = 5.18; *p* = 0.001). Group B exhibited much lower locomotor activity (7.3 ± 3.0 m) compared with the other groups, where mean distances ranged from 12.7 to 14.6 m. Post hoc comparisons confirmed that group B rats traveled significantly shorter distances than the animals from groups A, C, D, E, F, and G (Tukey HSD, *p* < 0.05 for each). No significant differences in distance were observed among the remaining groups. Open-Field Test outcomes are presented in Figure 4A,B.

### 3.4. Eight-Arm Radial Maze

**Figure 5:**
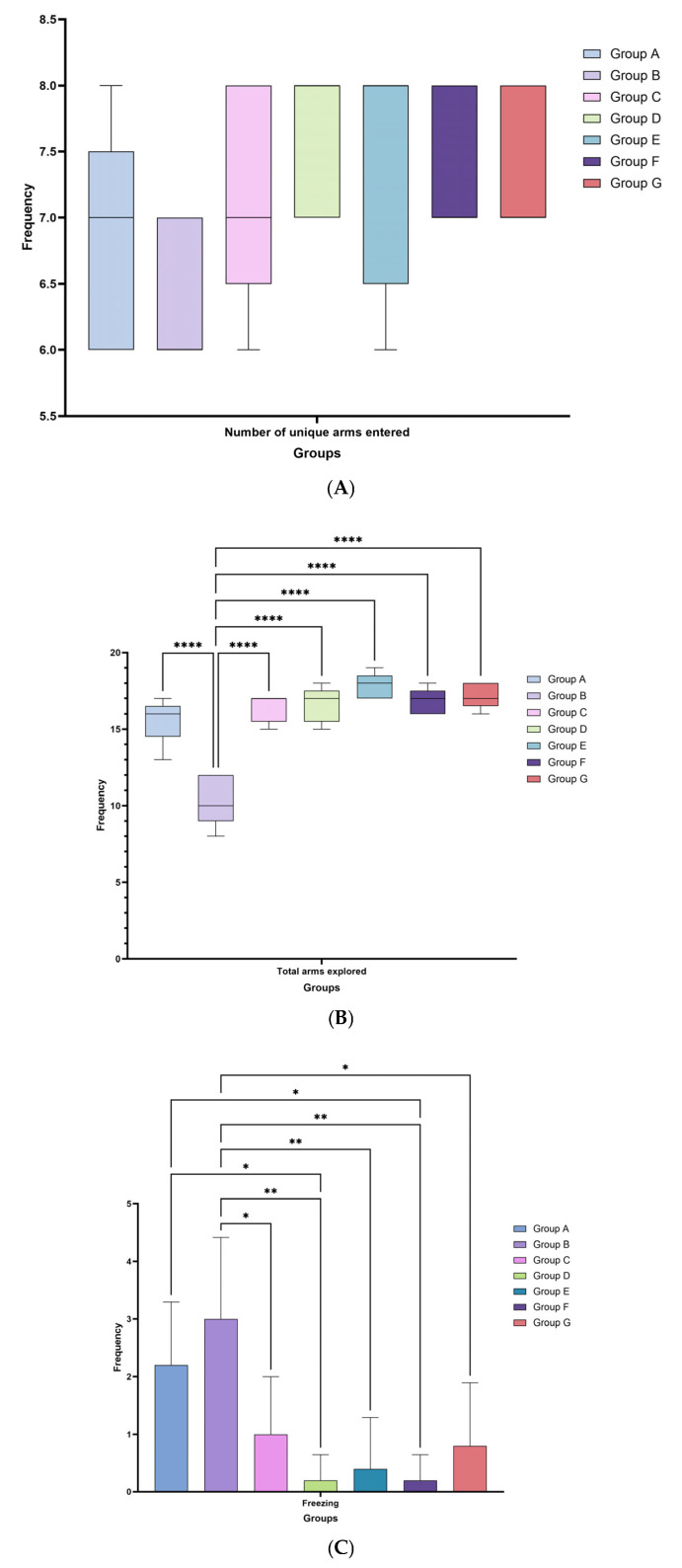

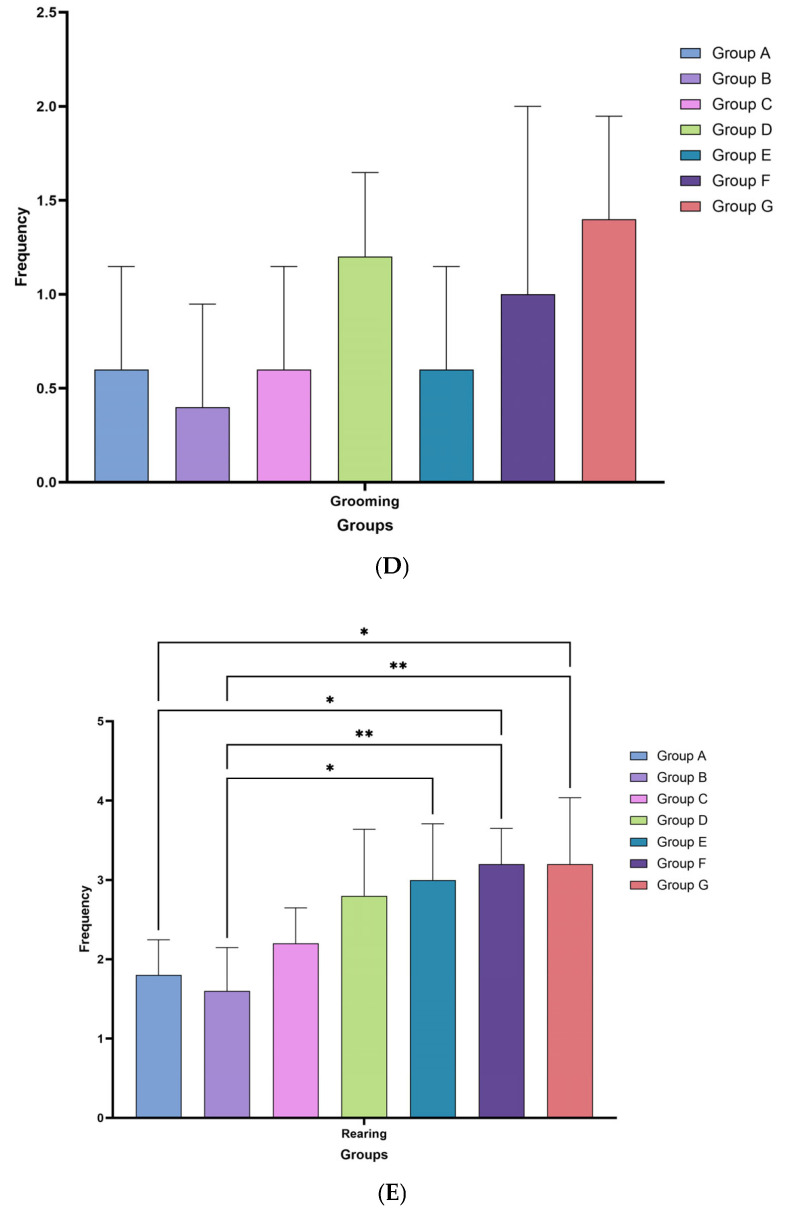
Eight-Arm Radial Maze performance and associated behaviors in adult female Wistar rats across dietary groups A–G (mean ± SD): (**A**) number of unique arms entered, (**B**) total arm entries, (**C**) freezing, (**D**) grooming, and (**E**) rearing. Legend: (**A**,**B**) In box-and-whisker plots, the central line indicates the median, boxes represent the interquartile range (25th–75th percentiles), and whiskers denote the data range (up to 1.5 × IQR). (**C**–**E**) Columns represent mean ± SD values. * *p* < 0.05; ** *p* < 0.01; **** *p* < 0.0001. Statistical significance was determined using Tukey’s HSD post hoc test after analysis of variance; all possible pairwise comparisons between groups were tested. A—standard laboratory rat diet, B—poultry meal-based diet, C—25% *T. molitor* meal, D—30% *T. molitor* meal, E—35% *T. molitor* meal, F—40% *T. molitor* meal, and G—45% *T. molitor* meal.

The number of unique arms entered by rats did not differ significantly between groups (df = 6; F = 1.84; *p* = 0.127). On average, rats in all groups explored approximately 6–8 unique arms (out of 8), indicating no significant group effect on initial arm exploration. A highly significant group effect was observed for the total number of arm entries, including repeated visits (df = 6; F = 23.30; *p* < 0.001). Group B made significantly fewer total entries (10.4 ± 1.67 entries) than any other group. Tukey’s post hoc tests confirmed that the total number of entries in group B was significantly lower than in the remaining groups (*p* < 0.001 for each comparison). No other pairwise differences in the total number of entries reached statistical significance. Freezing behavior during the maze test differed significantly between groups (df = 6; F = 6.21, *p* < 0.001). Group B exhibited the highest frequency of freezing episodes (3.0 ± 1.41), which was significantly greater than in groups C (1.0 ± 1.00), D (0.2 ± 0.45), E (0.4 ± 0.89), F (0.2 ± 0.45), and G (0.8 ± 1.10; Tukey HSD, *p* < 0.05 for each comparison with group B). The number of freezing episodes was also higher in group A (2.2 ± 1.10) than in groups D and F (*p* < 0.05). There were no significant differences between groups in grooming behavior (df = 6; F = 1.80, *p* = 0.135). The frequency of grooming behavior was low and comparable in all groups (generally 0–2 grooming episodes) during the maze trial. The frequency of rearing behavior differed significantly between groups (df = 6; F = 5.62, *p* < 0.001). Groups F and G showed the highest frequencies of rearing (3.2 ± 0.45 and 3.2 ± 0.84, respectively), both significantly greater than the lower levels observed in group A (1.8 ± 0.45) and group B (1.6 ± 0.55; Tukey HSD, *p* < 0.05 for all comparisons). In addition, group E (3.0 ± 0.71) reared significantly more frequently than group B (*p* < 0.05). No other significant differences in rearing behavior were found between the other groups. Eight-Arm Radial Maze performance and associated behaviors are presented in Figure 5A–E.

### 3.5. Nutritional Choice Test

**Figure 6:**
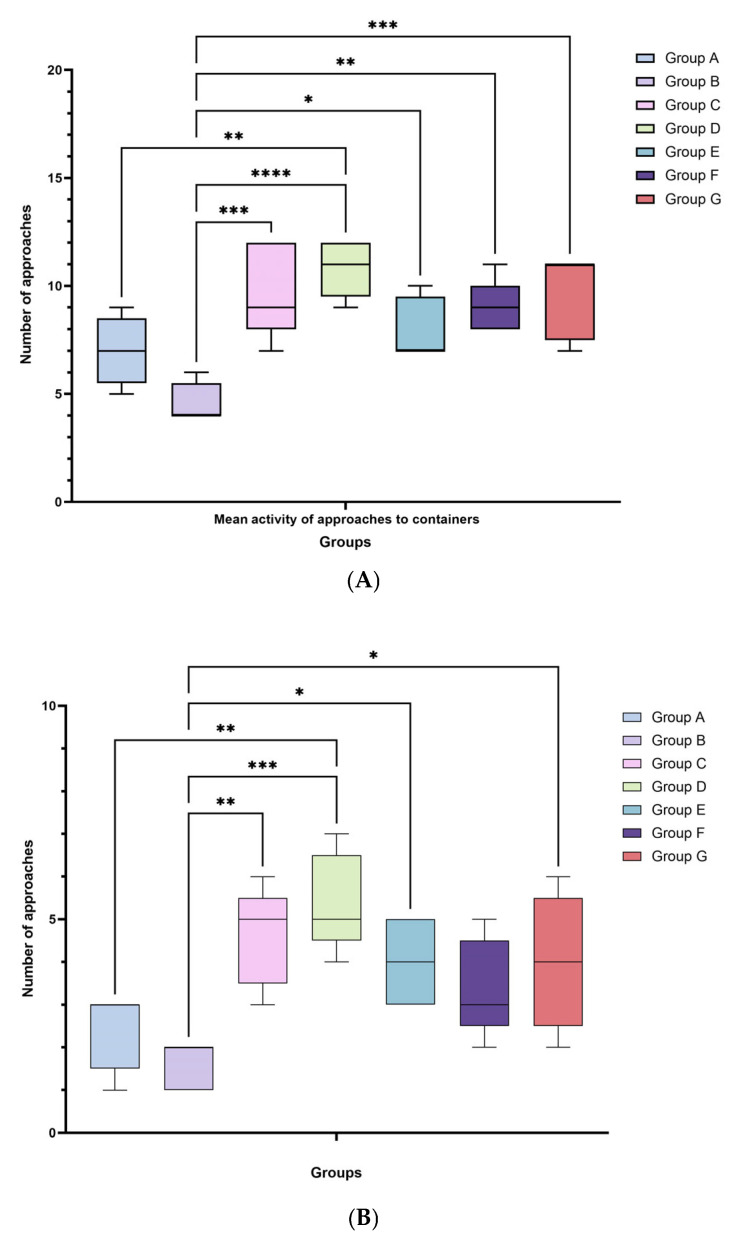

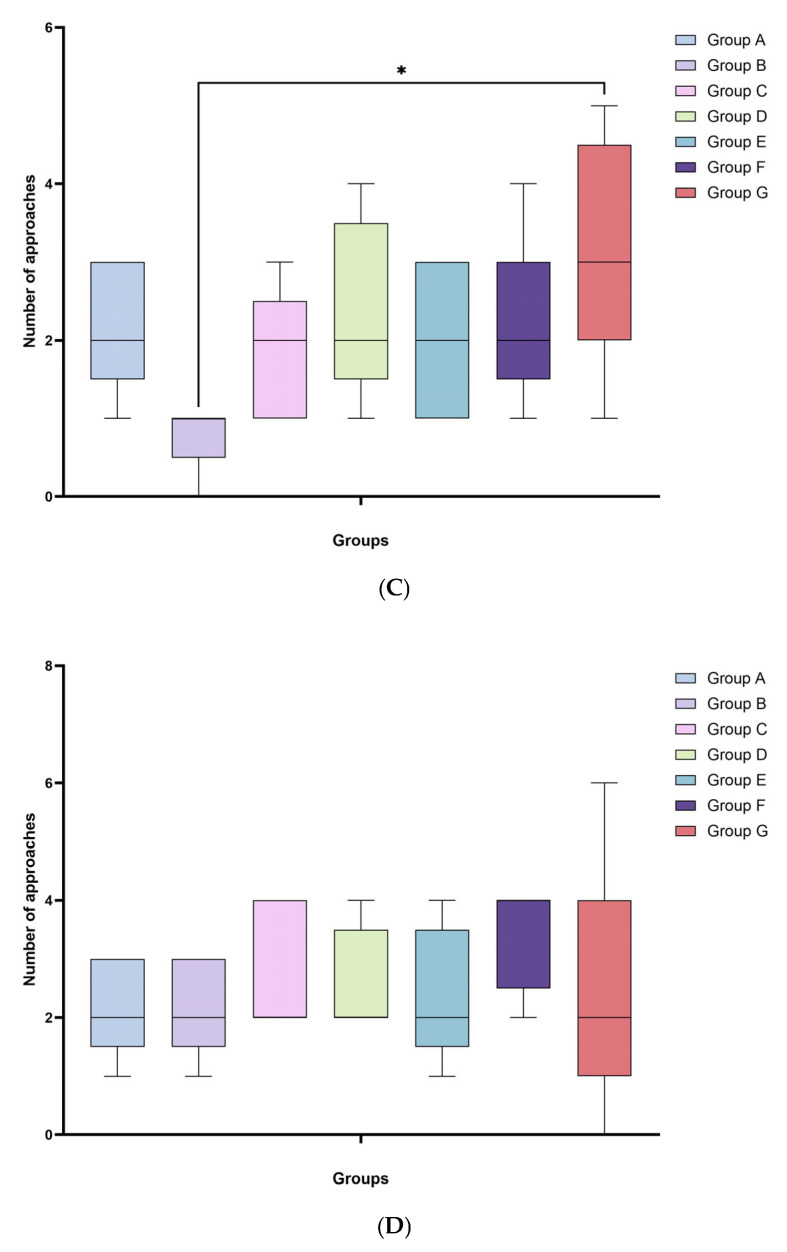
Total number of approaches to all feed sources and approaches to each simultaneously presented diet: (**A**) mean activity of approaches to containers, (**B**) insect-based (R), (**C**) poultry meal-based (P), and (**D**) standard laboratory diet (S) across groups. Legend: (**A**–**D**) In box-and-whisker plots, the central line indicates the median, boxes represent the interquartile range (25th–75th percentiles), and whiskers denote the data range (up to 1.5 × IQR). * *p* < 0.05; ** *p* < 0.01; *** *p* < 0.001; **** *p* < 0.0001. Statistical significance was determined using Tukey’s HSD post hoc test after analysis of variance; all possible pairwise comparisons between groups were tested. A—standard laboratory rat diet, B—poultry meal-based diet, C—25% *T. molitor* meal, D—30% *T. molitor* meal, E—35% *T. molitor* meal, F—40% *T. molitor* meal, and G—45% *T. molitor* meal. Annotation: In the nutritional choice test, group A and group B were administered feed containing 35% *Tenebrio molitor* meal.

To further explore feeding motivation and diet preference, a nutritional choice test was conducted. The overall number of feeding approaches differed significantly between groups (df = 6; F = 8.95; *p* < 0.001). Group B made the fewest approaches to food sources (4.6 ± 0.89 approaches), significantly fewer than groups C (9.8 ± 2.17), D (10.8 ± 1.30), E (8.0 ± 1.41), F (9.0 ± 1.22), and G (9.6 ± 1.95) (Tukey HSD, *p* < 0.05 for each comparison with group B). Group D, which showed the highest overall number of approaches, also made significantly more approaches than group A (7.0 ± 1.58; *p* < 0.05). A significant group effect was observed for approaches to the insect-based diet (df = 6; F = 6.89, *p* < 0.001). Group D exhibited the highest number of approaches to the R diet (5.4 ± 1.14), which was significantly greater than in group A (2.4 ± 0.89; *p* < 0.01). In contrast, group B approached the insect-based diet least frequently (1.6 ± 0.55), significantly less frequently than groups C (4.6 ± 1.14), D, E (4.0 ± 1.00), and G (4.0 ± 1.58) (*p* < 0.05 for each comparison with group B). Differences between groups in approaches to the poultry-based diet reached moderate significance (df = 6; F = 2.48; *p* = 0.047). Group G showed the highest number of approaches to the P diet (3.2 ± 1.48), which was significantly greater than in group B (0.8 ± 0.45; *p* < 0.05). No other significant pairwise differences between groups were identified in the number of approaches to the P diet. No significant group effect was observed for approaches to the standard diet (df = 6; F = 0.61, *p* = 0.721). All groups approached the S diet at comparable frequencies (approx. 2–3 attempts per animal), indicating no differences in preference for the standard diet for laboratory rats. Data are presented in Figure 6A–D.

## 4. Discussion

### 4.1. Clinical Parameters

One of the main concerns in the formulation of insect-rich diets is whether they remain nutritionally adequate and free from adverse effects. The present results suggest that *T. molitor* meal may replace a significant portion of conventional protein sources without compromising nutritional value or basic clinical parameters. All insect-fed groups maintained or improved their body weight and condition, and none exhibited clinical signs of nutrient deficiencies, such as lethargy or anemia. This indicates that *T. molitor* meal might provide sufficient nutrients to meet the rats’ requirements over the 60-day feeding period. Edible insects are recognized as a rich source of protein and micronutrients. Yellow mealworms, in particular, contain high-quality protein and lipids [20,35,42] and are widely regarded as a nutrient-dense feed and feed ingredients [43]. The dietary formulations used in this study likely benefited from these properties, as evidenced by the normal growth performance and physiological status of the insect-fed rats. Importantly, no evidence of overt organ dysfunction was detected under study conditions based on the clinical, hematological, and serum biochemical indicators measured. No deaths or morbidity occurred during the study. These results are consistent with previous toxicological assessments of *T. molitor*. Han et al. [44] conducted a rigorous 28-day subchronic toxicity test using lyophilized *T. molitor* powder in Sprague-Dawley rats and reported no adverse effects even at the highest dose of 3000 mg/kg body weight per day. In the cited study, the no-observed-adverse-effect level (NOAEL) was established at >3000 mg/kg/day [45]. This intake level is extremely high relative to body weight and substantially exceeds dietary exposure in typical feeding trials, indicating that *T. molitor* has a wide margin of safety. Similarly, a 70-day feeding study reported no toxicity or organ damage in rats fed *T. molitor*, further suggesting their safety as a novel protein source [46]. Regulatory assessments of dried *T. molitor* as a novel food have likewise concluded that yellow mealworms do not pose a safety concern when produced according to good hygiene practices [9,10]. No allergic or pathological responses were identified in the present study, and no feed refusal or behavioral signs of aversion were observed. The rats readily consumed insect-based diets during the entire experiment, suggesting their good palatability and tolerance.

Interestingly, a moderate negative correlation was observed between individual feed intake and body weight in some groups (B, C, D, F). This implies that in weeks when a rat consumed more feed, body weight tended to increase less or even decreased slightly by 1 or 2 g, and vice versa. Although this may appear counterintuitive, it likely reflects normal homeostatic regulation. As animals approach a higher body weight set point, compensatory adjustments in intake may occur to limit further weight gain. In turn, increased consumption may be observed after a small reduction in body weight. Such feedback mechanisms have been well documented in rodents and are not specific to insect-based diets. This pattern suggests that rats self-regulated their energy balance to some extent, even when fed energy-rich diets. In groups where no significant correlations were observed (A, E, G), feed intake was highly stable (A) or more variable without a clear linear relationship. Biologically, this association reflected within-animal week-to-week variation, indicating that periods of slightly increased feed intake were not necessarily associated with immediate weight gain, whereas small, transient decreases in body weight could be compensatory. In adult female Wistar rats, this pattern may reflect short-term homeostatic regulation of energy balance, including normal fluctuations in gut fill, hydration status, and physiological reproductive cycles, rather than impaired nutrient utilization. Importantly, this finding was not accompanied by sustained weight loss, reinduced physical conditioning, feed refusal, or other clinical signs of intolerance. Therefore, the negative correlation is best interpreted as evidence of adaptive regulation of intake and physiological state rather than a negative response to insect-based diets.

A comparison of the high-inclusion approach with the levels reported in the diets of other species provides valuable insights. In monogastric animals such as pigs and poultry, insect meals have been used as alternative protein sources with promising results. For example, complete replacement of fish meal protein with approximately 18.5% soldier fly larvae meal in pig diets had no negative impact on growth rates or blood parameters [47]. Some studies have even reported improvements in feed conversion and health indicators after insect inclusion [20,48,49]. Similarly, *T. molitor* meal has been incorporated at up to 25–50% to chicken and fish diets without adverse effects on growth performance [20]. The results reported across mammals, birds, and fish support the view that insects are important and safe feed ingredients. In this study, it was found that the inclusion of yellow mealworm meal in the diet in an amount of up to 45% was not associated with visible pathological changes in rats under experimental conditions. Such high inclusion levels could be expected to increase the risk of nutrient imbalance or decreased palatability, but the present data suggest that these thresholds were not exceeded. Although practical long-term formulations may not require such high inclusion levels, the high tolerance limit supports the view that insects might be used as primary protein sources in animal nutrition.

Previous studies have shown that insect proteins are generally well digested by rats, with protein utilization efficiency comparable to that of soy or fish meal [50]. However, some amino acids can be limiting in insect meals [32,51]. If not properly adjusted, these limitations could restrict growth or induce compensatory feeding behavior aimed at correcting nutrient deficiencies. Insect protein digestibility is typically high, but it may be somewhat lower than that of purified animal proteins due to the presence of chitin [52,53]. Sclerotin could also influence digestibility, but its effects have not been investigated to date. However, this constraint is mitigated by the fact that *T. molitor* larvae are soft-bodied and contain relatively low levels of chitin. Other researchers have noted that the low chitin content of *T. molitor* larvae enhances nutrient digestibility without the need for chitin removal [49,54]. A similar mechanism may have occurred in the present study. Even at the 45% inclusion level of yellow mealworm meal, rats were able to extract sufficient nutrients from feed. Although an accompanying microbiological study in rats fed 35% *T. molitor* meal suggested enrichment of bacterial taxa potentially involved in protein and chitin utilization [9], such adaptive mechanisms were not directly assessed in the present study. From a mechanistic perspective, the combination of a favorable amino acid profile and adaptive digestive responses may help explain why insect-rich diets supported growth comparably to conventional feeds.

Although diarrhea occurrence was limited in frequency and transient, its presence in the 45% inclusion group suggests that this highest dietary level may approach the upper threshold of gastrointestinal tolerance under the present experimental conditions. This finding should be considered when interpreting practical inclusion limits of *Tenebrio molitor* meal in rat diets. It is worth mentioning that chitin is not digested by host enzymes; it acts as dietary fiber and may exert metabolic effects. One possible explanation for the mild, transient diarrhea in group G could be the higher chitin intake associated with the 45% inclusion level of insect meal. Excess intake of indigestible fiber may accelerate intestinal transit or disturb water balance in the large intestine. However, in the current study, this effect was small and transient beyond a certain threshold. Importantly, chitin and its derivatives, such as chitosan and glycosaminoglycans, have been reported to exert beneficial health effects in moderate amounts [55,56,57]. A systematic review found that low dietary doses of chitin or chitosan reduced body weight gain and fat deposition by several percent and lowered cholesterol and serum glucose levels [58]. By binding fats and bile acids, chitin reduces lipid absorption and modulates gut hormones [57,59,60]. In addition, Chitin may influence host physiology by interacting with intestinal microorganisms and by modulating innate and adaptive immune responses [34,61,62,63]. In the present study, insect-rich diets consistently provided significant amounts of chitin. This may have contributed to the absence of hypercholesterolemia or excessive adiposity in insect-fed rats, potentially offsetting the high lipid content of the diets by preventing full lipid absorption. Although serum cholesterol and triglyceride concentrations did not differ significantly among groups in the present experiment, lipid-lowering effects of insect-based diets have been reported in previous studies. For example, obese rats fed *T. molitor* protein showed reduced plasma triglycerides and liver fat concentrations compared with control and reference groups [64]. Notably, group E (35% insect inclusion) exhibited the greatest weight gain without evidence of metabolic disturbance or extreme adiposity. This may suggest that chitin plays a balancing role. By promoting additional feed intake to support growth, chitin prevents pathological weight gain through its fiber-like properties. Similar observations were made by Kim et al. [65] and Kipkoech [62]. Deacetylated chitin derivatives had no effect on feed intake or body weight gain, but reduced abdominal fat deposition, likely by reducing lipase activity and fat absorption [66]. Furthermore, chitin can stimulate the gut immune system by acting as a mild prebiotic or immunomodulator. Metagenomic analyses revealed stable microbial diversity and increased chitinase activity in the gut of insect-fed rats, indicating that adaptive responses enhance chitin utilization [13]. Thus, from a mechanistic perspective, high dietary levels of chitin may slightly reduce digestible energy, potentially contributing to compensatory intake adjustments or mild diarrhea. At the same time, chitin is a source of functional fiber that may improve metabolic health.

The observed differences in feed intake may be partly attributable to differences in diet palatability. Insect-based pellets had a distinct, rich aroma compared with the standard laboratory feed, which may have increased their appeal for rats, at least initially. Mammals, including rats, are known to have a preference for feeds rich in umami and fat [67,68,69]. Insect meals naturally contain umami compounds and lipids that can enhance flavor [69,70]. The contrast between the modest consumption in group A and the higher intake in the remaining groups suggests that the inclusion of insect or animal meal significantly improved the palatability or acceptability of the diet. In insect-fed rats, intake was particularly high in the first week, likely due to the novelty or palatability of the feed, followed by subsequent self-regulation. This pattern of initial hyperphagia followed by stabilization is commonly observed when animals are introduced to highly palatable high-protein diets [71]. The fact that feed intake eventually stabilized in groups B–G suggests that any hedonic effect was moderated by physiological feedback. Nevertheless, the higher cumulative intake in insect-fed groups, which was approximately twice that of the standard diet group, highlights that these diets were highly palatable and did not induce adverse effects or aversion under study conditions. Palatability may also partially explain the consistently high feed intake in group E. Although previous pet food studies have shown that increased insect meal can alter the physical properties of extruded diets [32]. The present study did not directly assess sensory characteristics or feed preferences in rats. Therefore, any interpretation linking differences in intake to texture, odor, or taste should be considered tentative.

The present findings also suggest the regulation of energy intake in insect-fed rats. According to the related formulation study [32], the five *T. molitor*-based diets were nutritionally comparable in energy density, with GE values of 452.14–453.58 kcal/100 g and ME values of 356.48–357.93 kcal/100 g. Crude protein content was also consistently high across all formulations (25.73–25.90% DM). Even with unlimited access to calorie-rich insect-based feed, the rats did not exhibit continuous weight gain; instead, their growth curves reached a plateau, and their BCS approached a normal asymptote. Thus, the observed biological differences were unlikely to result from major variation in caloric density and might be interpreted in light of other dietary characteristics, including physical properties and feed acceptance. This suggests that *T. molitor* meal did not disrupt satiety mechanisms and did not impair the animals’ ability to meet their nutritional needs in this study. Given its high protein content, insect meal probably enhanced satiety signals more effectively than carbohydrate-rich feed. High-protein diets are known to increase diet-induced thermogenesis and the release of satiety-related hormones, thereby limiting overconsumption. The mid-study decline in feed intake observed in high-inclusion groups may reflect the activation of these protein-driven satiety mechanisms [71]. Over time, rats adjusted their feed intake to growth-related energy demands; therefore, the observed feeding patterns could be influenced by the interaction between palatability, which initially increased intake, and protein-induced satiety, which limited overconsumption. From a mechanistic perspective, the optimization of protein and energy intake is consistent with the protein leverage hypothesis. A clear pattern emerged in the multigroup model encompassing normal to very high protein inclusion levels: when protein and fat were abundant, as in the insect-rich diets, rats ultimately adjusted their feed intake to meet protein needs without overconsumption. In contrast, intake remained low in the standard diet group, potentially reflecting the physical limitations of bulk/fiber or inferior palatability.

Other clinical and physiological parameters remained normal in all dietary groups, further supporting the safety and adequacy of insect-rich diets. No significant differences in heart rate were found between groups, and all rats exhibited age-appropriate heart rates with typical fluctuations over time that can be attributed to stress or activity rather than diet. Repeated-measures ANOVA indicated that diet type had no effect on heart rate or respiratory rate. It suggests that high insect inclusion levels did not cause cardiovascular stress or changes in baseline metabolism. This is consistent with reports indicating that insect-fed animals maintain normal behavior and physiological function. Segú et al. [36] observed no adverse changes in rat behavior, metabolism, or intestinal morphology after long-term feeding of *T. molitor* larval meal. In the present study, qualitative observations revealed that rats also exhibited normal activity, grooming behavior, and eye and coat condition throughout the experimental period, with no differences between groups under studied conditions. Respiratory rate fluctuations were not significantly correlated with heart rate in any group, suggesting that insect meal had no effect on cardiorespiratory coordination or stress responses, and promoted homeostasis.

More broadly, the observed responses were likely influenced not only by the level of *T. molitor* meal but also by qualitative differences in diet composition. First, yellow mealworm meal provides highly digestible protein with high biological value, although some amino acids can be limiting if formulas are not properly balanced. Second, insect meal provided lipids that could affect satiety, texture, and metabolic responses after ingestion, even when overall energy density remained stable. Third, the presence of chitin could slightly reduce nutrient digestibility and disrupt gastrointestinal function, although this effect was likely limited because yellow mealworm larvae are relatively soft and contain less structural fiber than many other insect meals. In the formulation study, the five extruded diets remained very similar in gross and metabolizable energy despite the increased proportion of *T. molitor*, whereas crude protein and fat content increased only marginally (as a result of variances in the nutritional composition of components) in all formulations, suggesting that the biological differences observed here were due to subtle differences in nutrient form, digestibility, pellet properties, and feed acceptance rather than large changes in caloric density alone.

### 4.2. Hematology and Serum Biochemistry

Most of the hematological and biochemical serum parameters were within physiological ranges in all groups. The dietary inclusion of *T. molitor* meal at up to 45% did not adversely affect erythrocyte parameters during the study. RBC count, hemoglobin, and hematocrit remained similar across all diets and were comparable to the values reported in other studies on insect-derived proteins. For example, mice fed diets containing 0–20% defatted *H. illucens* (BSFL) showed no changes in RBC, HGB, or HCT [72], and pigs fed 10% yellow mealworm protein exhibited an increase in RBC counts [73]. The slightly reduced RDW at 40% *T. molitor* inclusion has no known adverse implications and may simply reflect differences in dietary composition rather than a pathology under study conditions. Retic (%) varied slightly between groups, with the highest values noted in the reference group that received the poultry meal-based diet and the lowest at 40% *T. molitor* inclusion. However, all values were within normal ranges, suggesting normal erythropoietic turnover. Total WBC count, LYM, MONO, and BASO were not affected by insect meal inclusion, suggesting the absence of immunosuppression. Neutrophils showed only a minor dietary effect, with the highest values observed at 35% *T. molitor* inclusion and the lowest at 40% inclusion, although these differences were small. Interestingly, a previous rodent study reported only mild leukocytosis at 20% BSFL inclusion [72], which was attributed to the immunostimulatory properties of chitin. No signs of inflammation were observed in this study. Eosinophil counts remained very low in all groups, and mean values were only slightly higher in the standard diet group. Platelet counts were significantly lower in rats fed poultry meal than in insect-fed groups. In all *T. molitor*-fed groups, PLT levels were within normal ranges and were comparable to those observed in group A, whereas group B was clearly thrombocytopenic. A similar pattern was reported in the literature, suggesting that insect protein is hemostatically neutral. In the work of Aryani et al. [72], 15–20% defatted BSFL inclusion induced only non-significant changes in PLT counts, which were highest in the control group and lowest in the group with moderate BSFL inclusion, without evidence of coagulopathy. These findings indicate that insect-based diets do not impair PLT production. In the present experiment, PLT counts were lowest in the insect-free diet. The low PLT counts in group B suggest that this reduction was more likely related to specific feed components, such as poultry meal, rather than to insect protein inclusion. Most serum biochemical parameters were not affected by *T. molitor* supplementation. ALT and ALP activity were within normal ranges in all groups. Many studies have similarly reported no increase in ALT or AST following insect protein inclusion. For example, mice fed diets containing up to 20% BSFL showed no significant changes in ALT or AST [72]. In the present study, AST was higher in the groups with the highest *T. molitor* inclusion (40–45%), whereas ALT remained unchanged. Since ALT is liver-specific, an isolated increase in AST may reflect extrahepatic effects, rather than hepatotoxicity. All AST values were within the species-specific reference range, and a histopathological examination would be required to confirm any hepatic effects. Serum CREA concentrations were slightly higher in rats fed insect meal than in the control group, although values remained within normal limits. Differences in BUN were minor. This pattern likely reflects increased dietary protein intake as high-protein diets are known to elevate CREA through increased protein catabolism rather than renal dysfunction. Studies in healthy humans have also shown higher CREA levels with high-protein diets, without any signs of impaired renal clearance [74]. Therefore, the slight increase in CREA is not unexpected and is unlikely to reflect pathology. Glucose, TG, and CHOL concentrations did not differ significantly between groups, suggesting that *T. molitor* meal itself does not alter the basal metabolic rate. In turn, specialized models of obese mice demonstrated that the replacement of casein with yellow mealworm protein improved blood lipid profiles [75].

Although several hematological and biochemical parameters differed significantly between groups, their biological significance appears limited, as observed changes were not accompanied by consistent or concordant changes in related markers within the same functional systems. For example, minor variations in red blood cell indices were not associated with changes in red blood cell count, hemoglobin, or hematocrit, and differences in liver or kidney parameters were not supported by concomitant abnormalities or clinical findings. Although statistically significant differences were found between groups for AST activity, all mean values were within established physiological ranges for rats, indicating that these changes might be interpreted as adaptive biochemical variation rather than evidence of clinically significant liver dysfunction. A similar pattern was observed for creatinine, where differences between dietary groups were present but values were within normal limits, suggesting preserved renal function.

Elevated serum urea concentrations may reflect increased amino acid catabolism associated with higher protein intake rather than organ dysfunction, particularly as other hepatic and renal indicators remained within physiological ranges. However, BUN values were unexpectedly lower than the reference range. This apparent discrepancy is most likely due to methodological and preanalytical factors, including differences in assay calibration, sample age, storage time prior to analysis, or temperature-related changes in serum enzymatic activity. Importantly, since creatinine, ALT, and AST were within physiological ranges, the overall biochemical profile might not indicate renal or hepatic impairment. Therefore, these findings are best interpreted as reflecting subtle physiological or nutritional changes.

### 4.3. Behavioral Test

The behavioral findings suggest that inclusion of *T. molitor* meal up to 45% did not adversely affect spontaneous locomotion or exploratory performance in rats under study conditions. However, the observed differences in open-field distance traveled, central-zone activity, and radial-maze arm entries should not be interpreted exclusively in terms of anxiety-like behavior. These endpoints may also reflect differences in general activity, arousal, motivational state, or nutritional/metabolic adaptation, particularly because the animals differed in feed intake and body-weight dynamics and were tested after an overnight fast. In this context, the reduced activity observed in the poultry-fed group may indicate lower locomotor or exploratory drive. Likewise, the maintained or increased activity observed in the insect-fed groups may reflect adequate nutritional support and behavioral engagement, although the present design does not allow firm conclusions regarding a specific anxiolytic effect. In the open-field test, all *T. molitor*-fed groups demonstrated increased locomotor activity and exploratory behavior comparable to the standard diet control group. In contrast, the poultry-fed group traveled significantly shorter distances and was less likely to explore the central zone under studied conditions. Similarly, in the eight-arm radial maze, the number of unique arm entries was similar across groups (6–8 arms on average), but the total number of arm entries was significantly higher in all insect-fed groups than in the poultry-fed group, indicating more frequent exploration. Notably, higher insect meal inclusion levels (35–45%) were associated with increased vertical rearing behavior compared with the poultry diet group, which may suggest enhanced exploratory behavior. Meanwhile, grooming frequency remained low and similar across all groups. Anxiety-related indicators, including central zone activity and the number of freezing episodes, were largely unaffected in *T. molitor*- fed groups. The only significant increase in anxiety-related behaviors was observed in the poultry-fed group, which spent less time in the central zone and displayed more frequent freezing episodes than most insect-fed groups. However, the inclusion of yellow mealworm meal did not increase freezing behavior and did not reduce locomotor activity compared with the standard laboratory diet in this experiment. Overall, rats receiving *T. molitor* diets displayed equal levels of locomotor activity and exploratory behavior to the control group, and the poultry diet was consistently associated with reduced activity in the nutritional choice test. Insect-fed rats also demonstrated greater feed-related exploratory activity, and groups C–G made significantly more approaches to feed sources than the poultry-fed group under study conditions. Rats habituated to edible insects approached the insect-based diet significantly more often than rats fed the poultry-based diet, whereas the number of approaches to the standard diet was the same across groups. The overall number of approaches was highest in group D (30% *T. molitor* meal inclusion), and insect-fed groups approached the insect-based diet significantly more often than the poultry-fed group. These findings might indicate that the *T. molitor*-based diet was highly palatable and motivating. Rodents are known to show strong preference for *T. molitor* over conventional treats [76], which is consistent with the present observation that insect-fed rats readily explored and selected the insect-based diet. In contrast, the low approach rates observed in poultry-fed rats may indicate reduced novelty or palatability of the offered diets. Because these tests were conducted after overnight fasting and primarily captured spontaneous locomotor and exploratory responses, the observed group differences may have been influenced by nutritional or metabolic state in addition to emotional reactivity, and lower activity observed in the poultry-fed group should not be attributed solely to potential anxiety-like behavior.

These behavioral results are consistent with reports that dietary protein source and composition influence activity and anxiety in rodents. In rats, high-protein diets have been associated with increased locomotor activity and arousal [77], which is consistent with the greater activity observed in insect-fed groups compared with the poultry-fed group. In contrast, diets high in saturated fat and sugar are frequently linked to anxiety-like behavior and reduced exploratory behavior in rodents [78]. No such impairments were observed in *T. molitor*-fed groups under study conditions, indicating that insect-based diets might not elicit the adverse effects typically associated with high-fat diets. These results suggest that even at high inclusion levels, insect-based diets supported normal exploratory behaviors.

**Figure 7:**
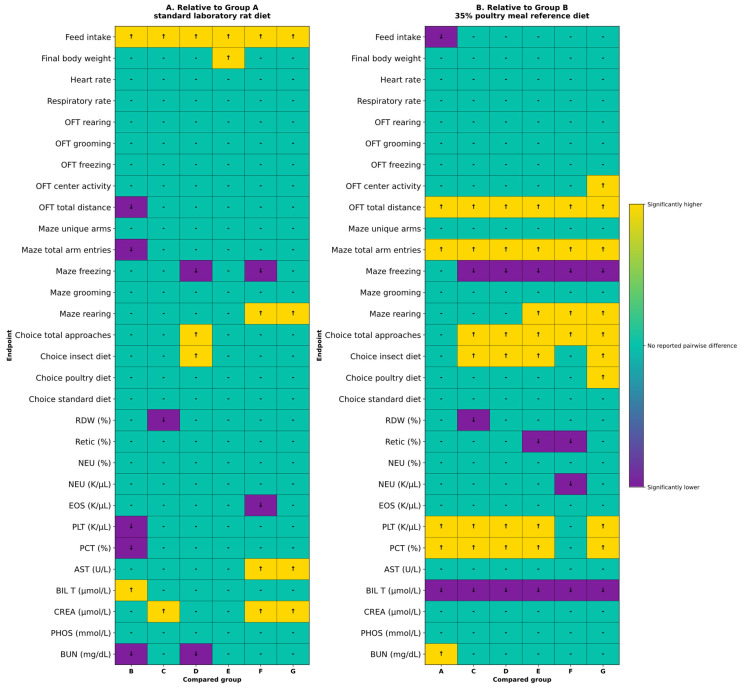
Qualitative heatmap summary of the statistically significant findings in adult female Wistar rats fed a diet containing 25–45% *Tenebrio molitor* meal. Legend: ↑—significantly higher than the A/B group; ↓—significantly lower than the A/B group; -—no reported significant pairwise difference; OFT—Open-Field Test; Maze—Eight-Arm Radial Maze; Choice—Nutritional Choice Test; RDW—Red Cell Distribution Width; Retic—reticulocytes; NEU—neutrophils; EOS—eosinophils; PLT—platelet count; PCT—plateletcrit; AST—aspartate aminotransferase; BIL T—total bilirubin; CREA—creatinine; PHOS—phosphorus; BUN—blood urea nitrogen. A—standard laboratory rat diet, B—poultry meal-based diet, C—25% *T. molitor* meal, D—30% *T. molitor* meal, E—35% *T. molitor* meal, F—40% *T. molitor* meal, and G—45% *T. molitor* meal; NEU (%) and PHOS only had an overall group effect but no specific Tukey HSD pairwise difference identified.

The statistically significant differences for measured parameters are further summarized in Figure 7. This summary indicates that the inclusion of *T. molitor* meal did not produce a uniform dose-dependent pattern across studied endpoints but resulted in selected parameter-specific differences. Most importantly, clinical and behavioral responses were generally maintained in the insect-fed groups, with the most pronounced behavioral differences observed compared with the reference group fed poultry meal.

### 4.4. Limitations

The presented study has certain limitations. Animals were fed ad libitum to allow for the assessment of voluntary feed intake and diet acceptance under conditions that reflect practical feeding scenarios. Although controlled feeding protocols can reduce variability, ad libitum access is commonly used in dietary tolerance studies and allows for the assessment of regulation of feed intake and palatability. However, it is important to note that this approach may introduce interindividual variability and partially account for the observed differences between groups. Another limitation is that all behavioral tests were performed on the same day, after an overnight fast. This fasting period was necessary because behavioral testing was performed immediately before euthanasia and postmortem assessment, and also to standardize the animals’ motivational state before the nutrient choice test. However, fasting may have influenced absolute measures of locomotor activity, exploratory behavior, food-driven motivation, and stress-related responses. Also, although overnight fasting standardized the motivational state, it may also have masked or modified true feeding behavior patterns under normal daily feeding conditions. Therefore, behavioral results should be interpreted primarily as comparative results between groups tested under identical conditions. It should be noted that the timing and structure of behavioral testing are valid methodological considerations that should be carefully weighed, and that a single-session design can be justified when the goal is to obtain comparable endpoint measurements under uniform conditions rather than multiple longitudinal behavioral readings [79]. The future studies should incorporate more sensitive behavioral tests or automated monitoring systems to detect subtle functional effects. The food choice test should be interpreted as an assessment of feed-directed exploratory behavior and relative interest in concurrently fed diets, not as a definitive test of palatability. Because the animals had previously had other exposure to the diet during the feeding trial, the observed approaches could have been influenced by familiarity, learned acceptance, or adaptation to the diet, in addition to palatability. Although the overall clinical, hematological, and biochemical findings do not indicate overt organ dysfunction under study conditions, the presence of statistically significant differences in selected biochemical markers (including AST and creatinine) suggests that potential subclinical effects cannot be ruled out and should be investigated in future studies The relatively small number of animals per group limits the statistical power of the study, particularly for behavioral and biochemical endpoints characterized by high interindividual variability. Power analysis indicated that the study design was adequate to detect large effects, but smaller, or moderate diet-related changes, including subtle linear or non-linear dose-dependent responses, may not have been detected. Therefore, the results should be interpreted as exploratory and should be verified in future studies with a larger sample size. Although Tukey’s HSD test was used to correct post hoc pairwise comparisons at each ANOVA, an additional global correction was not applied for all endpoints. Therefore, isolated statistically significant results may be associated with an increased residual risk of false positive interpretation. In this study, chitin content in the experimental diets was not directly quantified. However, it can be assumed that chitin levels increased with the inclusion of *T. molitor* meal in the diet. Because direct measurements of chitin content or its digestibility were not performed, its potential contribution to the observed effects remains unclear. Future studies should include direct quantification of chitin and its digestibility to better understand its role in insect-based diets.

The use of the rat as a biological model in this study should be interpreted as a preliminary step in assessing the safety margin/tolerance assessment of high concentrations of *T. molitor* meal, rather than as a direct simulation of feeding practice in the target species. This approach, in addition to the experiment duration, is consistent with the rationale for 90-day feeding studies within the OECD TG408 framework, which assess clinical signs of exposure, feed consumption, growth dynamics, hematology, serum biochemistry, organ function, and dose-dependent responses. However, the translation of these results to real-world feeding conditions in dogs and other monogastric species remains limited by interspecies physiological differences. As omnivores, the rat relies more heavily on cecal and colonic fermentation than carnivorous species, whereas in dogs and cats, hindgut fermentation plays a relatively minor role in nutrient recovery. This is particularly relevant when interpreting responses to the fiber-chitin fraction, protein digestibility, and nitrogenous metabolite production. Furthermore, amino acid metabolism is species-specific. Therefore, the absence of significant systemic abnormalities in rats cannot be considered equivalent to complete nutritional adequacy in companion animals. From a risk assessment perspective, it can therefore be cautiously concluded that the absence of serious adverse effects at such high dosage levels provides a useful toleration margin for lower, more commercially realistic levels. However, this remains a translational conclusion that requires confirmation in the target species. Therefore, future studies should primarily include palatability and preference studies in companion animals, gastrointestinal digestibility studies, and assessment of amino acid bioavailability, as well as long-term feeding studies that consider fecal quality, gut microbiome composition, clinical parameters, and amino acid balance.

## 5. Conclusions

The results of this study, supported by the existing literature, indicate that *T. molitor* meal is a nutritionally adequate, well tolerated under tested conditions, and effective protein source for monogastric animals, even at high dietary inclusion levels. The dose-dependent responses observed in feed intake and body composition highlight areas that warrant further investigation, including the potential limits of palatability and fiber tolerance at 45% inclusion or the optimal balance between insect protein and other nutrients to maximize growth performance. In this way, a general hypothesis postulating (a) a dose-response relationship, (b) changes in feed intake and behavior at higher inclusion levels, resulted in a complex, parameter-dependent answer: a dose-dependent response (particularly for groups E, F, and G) was seen in a few parameters, e.g., RDW and NEU. Most hematological parameters, however, did not change at all, which is a sign that diets containing *T. molitor* do not affect the animals adversely, as supported by physiological ranges of heart and respiratory rates. Rather, they induce metabolic changes that are coped with within physiological ranges, as hematology shows a series of adaptive processes. Testing insect-containing diets against a standard laboratory rat diet on one hand and a poultry diet in terms of behaviors showed that these diets by themselves improve corresponding results, rather than resulting in a dose-related response. Although the observed results were generally consistent, the relatively small sample size should be considered a limitation, especially for behavioral and biochemical endpoints, which are inherently variable. Therefore, these results should be interpreted with caution and verified in studies with greater statistical power to reduce the risk of type I and II errors. Future studies should also examine the long-term effects of insect-rich diets on organ health and aging, and mechanistic studies should explore protein digestion and changes in the gut microbiome. Nevertheless, the present findings support the feasibility of using *T. molitor* to formulate high-protein diets, with minimal modifications to incorporate its unique components, such as chitin. This study contributes to the growing body of evidence supporting insects as a sustainable protein source in animal nutrition. The observed weight gain patterns, absence of safety concerns, and physiological adaptability suggest that *T. molitor* meal may be incorporated into the diet at levels substantially exceeding traditional usage without compromising animal health or welfare. However, because no single dietary inclusion level consistently produced the best results for all physiological and behavioral parameters, this study does not support the existence of a single, universally optimal formula. Intermediate inclusion levels, particularly 30–35%, demonstrated beneficial responses in selected parameters such as body weight development and feed-oriented exploratory behavior, but these effects were not fully consistent across endpoints. Therefore, this range should be interpreted as a potentially promising area for further research. The 45% inclusion level is better considered an upper tolerance and safety margin under the conditions studied rather than a preferred, routine target.

## 6. Patent

The present study gave rise to patent P.443579—hypoallergenic dog food. The patent covers a hypoallergenic feed formulation for dogs suffering from food-responsive enteropathies. Hypoallergenic dog food contains *T. molitor* meal (inclusion level: 10% to 60%, preferably 35%), potato and/or sweet potato by-products (10% to 65%, preferably 48%), plant substrates of the plantain family (0.01% to 15%, preferably 7.3%), animal or vegetable fat (0.1–15%), herbs and minerals (0.01% to 5%, preferably 1.8%), berries (0.01% to 8%, preferably 0.6%), functional additives such as water- and fat-soluble vitamins (0.01% to 15%, preferably 6.3%), and plants of the flax family (0.01% to 5%, preferably 1%). The patented formulation has the following nutritional composition: crude protein—11% to 41% (preferably 25%); crude fat—3% to 27% (preferably 15%); crude ash—1% to 9% (preferably 5%); and crude fiber—1% to 9% (preferably 5%).

## Figures and Tables

**Table 1:** Hematological parameters (mean ± SD) in each group of rats with the corresponding ANOVA *p*-values.

Parameter	Reference Range	Group A (Mean ± SD)	Group B (Mean ± SD)	Group C (Mean ± SD)	Group D (Mean ± SD)	Group E (Mean ± SD)	Group F (Mean ± SD)	Group G (Mean ± SD)	*p*-Value
RBC (M/μL)	7.16–9.24	8.72 ± 0.29	8.90 ± 0.38	9.49 ± 0.57	9.06 ± 0.51	9.24 ± 0.24	8.85 ± 0.29	9.01 ± 0.84	0.246
HCT (%)	38.5–49.2	47.64 ± 1.37	49.50 ± 3.16	50.18 ± 3.37	48.50 ± 2.86	50.08 ± 2.00	46.88 ± 3.33	49.22 ± 5.43	0.640
HGB (g/dL)	13.7–17.2	15.44 ± 0.43	15.84 ± 0.73	16.18 ± 0.87	15.52 ± 0.76	15.70 ± 0.56	15.04 ± 0.90	15.78 ± 1.45	0.542
MCV (fL)	50.3–57	55.03 ± 0.97	55.82 ± 1.01	52.89 ± 1.88	53.66 ± 2.48	54.17 ± 1.34	53.89 ± 1.57	54.52 ± 2.23	0.061
MCH (pg)	17.6–20.3	17.69 ± 0.26	17.80 ± 0.37	17.07 ± 0.74	17.38 ± 0.53	17.38 ± 0.39	17.21 ± 0.50	17.55 ± 0.66	0.105
MCHC (g/dL)	33.2–37.8	32.15 ± 0.46	31.89 ± 0.61	32.27 ± 0.64	32.39 ± 0.66	32.11 ± 0.37	31.95 ± 0.35	32.18 ± 0.51	0.673
RDW (%)	10.6–14.6	20.40 ± 0.98	19.60 ± 0.54	18.46 ± 0.87	19.32 ± 1.10	19.62 ± 0.85	19.46 ± 0.98	19.20 ± 1.82	0.024 *
Retic (%)	1.4–3.9	3.90 ± 0.69	3.06 ± 0.40	3.54 ± 0.38	3.62 ± 0.70	3.18 ± 0.57	3.96 ± 0.89	4.12 ± 0.54	0.031 *
Retic (K/μL)	108.3–312.6	339.0 ± 51.2	280.6 ± 31.1	341.0 ± 31.0	337.4 ± 55.9	298.3 ± 46.2	351.9 ± 66.4	357.9 ± 34.5	0.067
WBC (K/μL)	0.96–7.88	7.78 ± 1.72	8.15 ± 1.04	8.56 ± 1.16	7.38 ± 1.24	7.94 ± 0.95	8.86 ± 1.32	8.82 ± 0.97	0.587
NEU (%)	8.8–43.8	18.00 ± 6.91	24.40 ± 6.50	21.80 ± 6.53	15.40 ± 6.15	21.40 ± 7.86	18.20 ± 5.54	15.80 ± 4.55	0.039 *
LYM (%)	48.9–88.1	78.06 ± 7.58	70.12 ± 6.80	72.36 ± 7.46	79.28 ± 6.41	73.70 ± 8.04	77.06 ± 5.52	80.14 ± 4.59	0.079
MONO (%)	1–3.6	2.86 ± 1.07	2.70 ± 0.76	2.62 ± 0.38	2.40 ± 0.74	3.12 ± 0.64	2.32 ± 0.75	2.60 ± 0.74	0.695
EOS (%)	0.3–4.7	1.06 ± 0.68	2.20 ± 2.87	1.30 ± 0.84	2.12 ± 2.22	1.08 ± 0.66	1.14 ± 0.75	1.02 ± 0.83	0.446
BASO (%)	0–0.7	4.14 ± 6.85	4.64 ± 6.15	3.22 ± 3.01	3.08 ± 4.02	0.54 ± 0.64	2.44 ± 3.95	4.36 ± 4.23	0.852
NEU (K/μL)	0.15–1.11	1.21 ± 0.58	1.90 ± 0.76	1.67 ± 0.59	0.95 ± 0.43	1.69 ± 0.49	1.61 ± 0.53	1.34 ± 0.41	0.023 *
LYM (K/μL)	0.68–6.8	6.13 ± 1.56	5.59 ± 0.94	6.11 ± 1.35	6.02 ± 1.08	5.78 ± 0.84	6.75 ± 1.37	7.02 ± 0.86	0.603
MONO (K/μL)	0.01–0.13	0.20 ± 0.30	0.20 ± 0.05	0.23 ± 0.08	0.18 ± 0.05	0.25 ± 0.05	0.17 ± 0.07	0.17 ± 0.06	0.812
EOS (K/μL)	0.01–0.14	0.20 ± 0.17	0.04 ± 0.02	0.03 ± 0.02	0.04 ± 0.01	0.03 ± 0.02	0.02 ± 0.01	0.03 ± 0.01	0.0006 *
BASO (K/μL)	0–0.02	0.13 ± 0.10	0.05 ± 0.06	0.04 ± 0.03	0.03 ± 0.05	0.03 ± 0.02	0.02 ± 0.04	0.04 ± 0.04	0.086
PLT (K/μL)	599–1144	625.0 ± 112.0	535.2 ± 49.1	708.6 ± 141.6	644.0 ± 70.5	769.6 ± 71.8	681.4 ± 63.5	680.4 ± 100.0	0.041 *
MPV (fL)	6.4–9.5	8.34 ± 0.61	8.14 ± 0.25	8.72 ± 0.60	8.22 ± 0.36	8.40 ± 0.46	8.26 ± 0.36	8.34 ± 0.31	0.311
PDW (fL)	46.8–68.5	9.74 ± 0.80	9.62 ± 0.28	9.34 ± 0.46	9.48 ± 0.39	9.72 ± 0.43	9.40 ± 0.58	9.78 ± 0.89	0.751
PCT (%)		0.52 ± 0.08	0.41 ± 0.03	0.61 ± 0.13	0.54 ± 0.06	0.65 ± 0.05	0.56 ± 0.05	0.57 ± 0.09	0.033 *

Legend: A—standard laboratory rat diet, B—poultry meal-based diet, C—25% *T. molitor* meal, D—30% *T. molitor* meal, E—35% *T. molitor* meal, F—40% *T. molitor* meal, and G—45% *T. molitor* meal. RBC—Red Blood Cell count; HCT—Hematocrit; HGB—Hemoglobin; MCV—Mean Corpuscular Volume; MCH—Mean Corpuscular Hemoglobin; MCHC—Mean Corpuscular Hemoglobin Concentration; RDW—Red Cell Distribution Width; Retic (%)—Reticulocyte percentage; Retic (K/μL)—Absolute reticulocyte count; WBC—White Blood Cell count; NEU (%)—Neutrophils; LYM (%)—Lymphocytes; MONO (%)—Monocytes; EOS (%)—Eosinophils; BASO (%)—Basophils; NEU (K/μL)—Absolute neutrophil count; LYM (K/μL)—Absolute lymphocyte count; MONO (K/μL)—Absolute monocyte count; EOS (K/μL)—Absolute eosinophil count; BASO (K/μL)—Absolute basophil count; PLT—Platelet count; MPV—Mean Platelet Volume; average size of platelets; PDW—Platelet Distribution Width; variation in platelet size; PCT (%)—Plateletcrit. *—Differences between groups are significant at *p* < 0.05.

**Table 2:** Serum biochemical parameters (mean ± SD) in each group of rats with the corresponding ANOVA *p*-values.

Parameter	Reference Range	Group A (Mean ± SD)	Group B (Mean ± SD)	Group C (Mean ± SD)	Group D (Mean ± SD)	Group E (Mean ± SD)	Group F (Mean ± SD)	Group G (Mean ± SD)	*p*-Value
Glucose (mmol/L)	4.94–9.05	4.06 ± 0.78	4.36 ± 0.89	4.76 ± 0.47	4.42 ± 0.55	4.52 ± 0.28	4.50 ± 0.34	4.80 ± 0.29	0.449
TG (mmol/L)	0.18–1.98	0.41 ± 0.08	0.38 ± 0.06	0.44 ± 0.11	0.42 ± 0.12	0.44 ± 0.15	0.44 ± 0.06	0.47 ± 0.12	0.859
ALT (U/L)	14–64	43.00 ± 5.48	46.60 ± 5.08	44.40 ± 3.78	44.20 ± 3.70	40.60 ± 2.70	41.00 ± 6.04	40.20 ± 5.40	0.285
AST (U/L)	64–222	110.00 ± 34.93	99.00 ± 21.13	81.20 ± 13.84	84.20 ± 19.24	85.20 ± 15.92	81.20 ± 18.41	77.00 ± 21.26	0.041 *
BIL T (μmol/L)	1.20–3.59	0.00 ± 0.00 ^#^	1.24 ± 2.77	0.00 ± 0.00 ^#^	0.00 ± 0.00 ^#^	0.00 ± 0.00 ^#^	0.00 ± 0.00 ^#^	0.00 ± 0.00 ^#^	0.037 *
ALP (U/L)	18–62	55.80 ± 19.06	63.60 ± 24.00	63.20 ± 16.47	57.20 ± 15.92	58.00 ± 19.04	64.20 ± 21.48	71.80 ± 36.16	0.944
CREA (μmol/L)	26.5–53.0	22.60 ± 3.85	27.20 ± 3.56	22.20 ± 2.17	24.20 ± 1.79	22.60 ± 2.07	23.00 ± 2.55	24.40 ± 2.88	0.035 *
UREA (mmol/L)	1.95–4.16	6.52 ± 0.68	7.22 ± 0.76	6.64 ± 0.93	7.14 ± 1.02	7.06 ± 0.66	6.58 ± 0.42	6.28 ± 0.80	0.280
ALB (g/L)	37–58	39.60 ± 1.34	39.20 ± 0.84	39.60 ± 1.52	39.80 ± 1.30	39.00 ± 1.00	38.60 ± 1.14	37.60 ± 1.82	0.279
CK (U/L)	218–1320	889.00 ± 191.83	829.20 ± 129.63	854.20 ± 324.69	829.60 ± 101.54	777.80 ± 173.98	894.00 ± 287.32	936.00 ± 235.99	0.917
CALC (mmol/L)	2.38–3.03	2.60 ± 0.05	2.56 ± 0.10	2.66 ± 0.05	2.60 ± 0.11	2.60 ± 0.07	2.58 ± 0.05	2.60 ± 0.07	0.448
PHOS (mmol/L)	1.46–3.07	1.58 ± 0.29	1.26 ± 0.24	1.40 ± 0.32	1.56 ± 0.34	1.62 ± 0.27	1.40 ± 0.27	1.64 ± 0.25	0.030 *
BUN (mg/dL)	11.7–25	3.40 ± 0.55	3.60 ± 0.89	3.60 ± 0.55	4.00 ± 0.71	3.40 ± 0.55	3.60 ± 0.55	3.20 ± 0.45	0.045 *
CHOL (mmol/L)	0.60–2.51	1.72 ± 0.43	1.68 ± 0.32	1.59 ± 0.30	1.80 ± 0.33	1.71 ± 0.25	1.52 ± 0.22	1.57 ± 0.42	0.898

Legend: A—standard laboratory rat diet, B—poultry meal-based diet, C—25% *T. molitor* meal, D—30% *T. molitor* meal, E—35% *T. molitor* meal, F—40% *T. molitor* meal, and G—45% *T. molitor* meal. Glucose—Serum sugar level; TG- Triglycerides; ALT—Alanine aminotransferase; AST—Aspartate aminotransferase; BIL T—Total bilirubin; ALP (U/L)—Alkaline phosphatase; CREA (μmol/L)—Creatinine; UREA—Urea; ALB—Albumin; CK—Creatine kinase; CALC—Calcium; PHOS—Phosphorus; BUN—Serum urea nitrogen; CHOL—Cholesterol. *—Differences are significant at *p* < 0.05. ^#^—Total bilirubin concentrations were at or below the lower detection limit of the analytical method. Therefore, values were reported as 0.0 in accordance with the analyzer output and should be interpreted as reflecting minimal circulating bilirubin.

## Data Availability

The original contributions presented in this study are included in the article/Supplementary Material. Further inquiries can be directed to the corresponding author.

## Supplementary Materials

The following supporting information can be downloaded at: https://www.mdpi.com/article/10.3390/ani16111623/s1. Table S1: Mean content (%) of crude protein, fat, ash, fiber, and carbohydrates on a dry matter basis in seven formulas used for adult female Wistar rats feeding. Reference [32] is cited in the Supplementary Materials.
