# Supplementary material for: Influence of Tenebrio molitor Meal Inclusion (25–45%) on Clinical and Behavioral Responses in Laboratory Rat Feeding Trial
Source: Animals (Basel). 2026 May 26;16(11):1623. doi: 10.3390/ani16111623 (PMC13255664; doi:10.3390/ani16111623)
Supplement: Supplementary file 1 [file animals-16-01623-s001.zip › animals-4316894-supplementary.pdf]

animals

Table S1. Mean content (%) of crude protein, fat, ash, fiber, and carbohydrates on a dry matter basis in seven formulas used for adult female Wistar rats feeding.

Formula	Crude Protein (%)	Crude Fat (%)	Crude Ash (%)	Crude Fiber (%)	Carbohydrates (%)
Standard laboratory rat diet	31.47 ± 1.78	6.01 ± 0.41	4.58 ± 0.49	5.01 ± 0.46	52.93 ± 2.17
Poultry meal 35%	25.93 ± 0.91	13.51 ± 0.74	5.68 ± 0.67	5.26 ± 0.54	42.99 ± 1.58
Insect meal 25%*	25.77 ± 0.92	13.56 ± 0.78	5.36 ± 0.55	5.16 ± 0.59	43.15 ± 1.46
Insect meal 30%*	25.73 ± 0.87	13.68 ± 0.68	5.41 ± 0.59	5.17 ± 0.65	43.01 ± 1.36
Insect meal 35%*	25.83 ± 0.78	13.79 ± 0.65	5.37 ± 0.43	5.22 ± 0.49	42.79 ± 1.04
Insect meal 40%*	25.75 ± 0.95	13.92 ± 0.68	5.42 ± 0.67	5.20 ± 0.57	42.71 ± 1.65
Insect meal 45%*	25.90 ± 1.34	14.07 ± 0.86	5.73 ± 0.83	5.29 ± 0.69	42.01 ± 2.04

Key: the presented values are means ± SD (n = 5 per formula). Annotation: insect meal was replaced with potato by-products or fish oil in the same proportions to maintain the original parameters. *- Data published in: Gałęcki, R.; Pszczółkowski, B.; Zielonka, Ł. Experiences in Formulating Insect-Based Feeds: Selected Physicochemical Properties of Dog Food Containing Yellow Mealworm Meal. Animals 2025, 15, 2087. https://doi.org/10.3390/ani15142087
